# The Case for Multifaceted, Integrative Motivation Interventions Embedded in Classrooms

**DOI:** 10.1007/s10648-026-10177-w

**Published:** 2026-06-11

**Authors:** Lisa Linnenbrink-Garcia, Allan Wigfield, Lauren Cabrera, Samuela Mouzaoir, David McKinney, Pei Pei Liu, Gwen Marchand, Jennifer A. Schmidt, Christopher J. Harris, John Guthrie

**Affiliations:** 1 Department of Counseling, Educational Psychology, and Special Education, Michigan State University, East Lansing, MI USA; 2 Department of Human Development and Quantitative Methodology, University of Maryland, College Park, College Park, MD USA; 3 Department of Education and the Brain & Motivation Research Institute (bMRI), Korea University, Seoul, Korea; 4 Hector Research Institute for Education Sciences and Psychology, University of Tubingen, Tubingen, Germany; 5 Department of Education, University of Michigan-Dearborn, Dearborn, MI USA; 6 Ingham Intermediate School District, Mason, MI USA; 7 Science and Engineering, WestEd, San Francisco, CA USA; 8 Education Department, Colby College, Waterville, ME USA; 9 Department of Educational Psychology, Leadership, and Higher Education, University of Nevada, Las Vegas, Las Vegas, NV USA

**Keywords:** Motivation interventions, Theoretical integration, Complex dynamic systems

## Abstract

Motivation scholars have grown increasingly interested in the application of motivation theory to educational practice. We provide an overview of various approaches to supporting students’ motivation and then focus specifically on the potential benefits of multifaceted interventions that integrate across theoretical perspectives and are embedded in classrooms to support students’ motivation. We also discuss how multifaceted interventions can be guided by an understanding of complex dynamic systems (CDS) and present a brief overview of how CDS can be applied to understanding motivation and education. We then discuss in detail two multifaceted, theoretically integrated motivation intervention programs embedded in classrooms that have been successfully implemented. The first focused on reading motivation and engagement and was implemented at both the elementary and middle school levels. The second focused on science motivation and engagement at the middle school level. We close with a discussion of the challenges and promises of implementing embedded motivation interventions that integrate across theories and use a CDS framework.

Over the last 15 years motivation researchers have conducted numerous theoretically based intervention studies designed to enhance students’ motivation and engagement (see Harackiewicz & Priniski, 2018; Hulleman & Barron, 2016; Linnenbrink-Garcia et al., 2016; Patall et al., 2026; Rosenzweig et al., 2022; for reviews of this work). Most of these have focused on a specific aspect of motivation, such as utility value. We (and others like Harackiewicz & Priniski, 2018; Hulleman & Barron, 2016; and Rosenzweig et al., 2022) call these “targeted” interventions because they seek to address a specific educational problem, such as increasing participation in STEM careers by women, and often target a single aspect of motivation. In addition, most (but not all) targeted motivation interventions are designed and implemented to change students’ motivation directly without changing to a great extent the underlying educational context students are experiencing. As we discuss below, this work has produced some impressive results. Yet, in this article we make the case that it is time to develop more multifaceted interventions that integrate across theories, are embedded in classroom contexts and involve working with classroom instructors, and consider motivation and classrooms as complex dynamic systems. We think this approach is particularly important for interventions done in K – 12 classrooms but also see potential in doing so at the college level as well.

To set the stage for this position, we first provide an overview of the motivation intervention field, relying on extant comprehensive reviews rather than doing a full review ourselves. We discuss what we see as the strengths of that work as well as its limitations. We then present our case for why we think intervention researchers should build on the prior motivation intervention research to conduct multifaceted, integrated, embedded interventions. We present both theoretical and practical reasons, arguing for the benefits of considering both motivation and classrooms as complex dynamic systems that require theoretically integrated, embedded interventions. To illustrate and support our case, we describe two extant motivation interventions, Concept Oriented Reading Instruction (CORI; Guthrie et al. 2004a, b: Guthrie et al., 2012) and Motivation-Planning Lessons to Activate eNgagement (M-PLANS; Harris et al., 2023; Marchand et al., 2022) to provide examples for researchers wanting to develop such interventions in the future. We discuss how each relied on and integrated across multiple theories and why, present each intervention’s approach to teacher professional development, describe how the programs were implemented in classroom settings, and summarize some key results concerning how the interventions affected teachers’ instructional practices and student outcomes. We close with a discussion of the challenges and promises of these types of intervention.

Before we begin, let us note two overarching points. First is how we connect to the special issue’s theme of integration across the four research areas discussed in the editors’ introductory article. We focus on theoretical integration within the motivation field rather than across the areas of motivation, metacognition, self-regulation, and personality because we think the state of knowledge about such integrations in the motivation field is not complete enough to make recommendations regarding interventions across some or all of the four other constructs discussed by authors of the articles in this special issue. Our article addresses one of the four primary questions the editors of the special issue posed in their call for paper proposals: *What kinds of interventions does the field need to enhance students’ motivation*,* metacognition*,* self-regulation*,* and personality*,* separately or together?* Second, we use the term “motivation interventions” broadly in this article. We include targeted, single construct approaches, multiple construct approaches, and work that changes classroom instructional practices to enhance motivation under the term interventions.^1^

## Current Approaches to Motivation Interventions

Motivational scholars have long been interested in the nuanced ways that motivation shapes students’ engagement, learning, and achievement (e.g., Weiner, 1990; Wigfield & Koenka, 2020). For the past several decades, motivational scholars have applied this research to educational settings by seeking to enhance students’ motivation in school using a variety of methods ranging from large-scale attempts to shift the climate and instructional practices in entire schools (e.g., Maehr & Midgley, 1996) to efforts to shift a specific form of motivation such as attributions, student mindsets, and task values (e.g., Harackiewicz et al., 2016; Wilson & Linville, 1982; Yeager et al., 2019). Several extensive reviews already exist summarizing this work (Lazowski & Hulleman, 2016; Harackiewicz & Priniski, 2018; Hulleman & Barron, 2016; Rosenzweig et al., 2022). Thus, rather than conducting another systematic review, we utilize the information presented in these reviews to help us build our case for why multifaceted interventions are needed. That is, we see the overall purpose of our article as conceptual rather than as a research review.

Lazowski and Hulleman (2016) conducted a meta-analysis of 74 published and unpublished intervention studies grounded in motivation theory that were conducted in educational contexts rather than in laboratory settings. They found that these field-based motivation interventions were generally successful, producing on average a medium overall effect size (0.49) across a variety of outcomes tested. Although they found that there was significant heterogeneity in the overall effect size, the only significant moderator identified was the type of experimental design, with quasi-experimental designs yielding larger effects than randomized designs. None of the other moderators, including the study’s theoretical framework, grade level, or type of dependent variable (self-report, performance, behavioral), explained the variation in the effect sizes. As we note later, this heterogeneity in findings may speak to the importance of understanding more fully how local contexts impact the effectiveness of the intervention (see Kaplan et al., 2020).

Of particular importance for our thesis in this paper was the “multiple theoretical frameworks” code used by Lazowski and Hulleman (2016), which was applied to 23 of the 92 effect sizes. The average effect size for this sub-type of studies was 0.41, suggesting a similar effect size to other types of interventions tested. However, in reviewing the studies that were coded as “multiple theoretical perspectives,” very few of these could be considered “theoretically integrated” in the way we discuss it below. Additionally, their review focused on field-based *experiments* and thus they limited their review to both randomized control trials and quasi-experimental designs; they did not include design-based implementation studies or other approaches that are more commonly used by scholars utilizing a Complex Dynamic Systems (CDS) framework.

Two narrative reviews (Hulleman & Barron, 2016; Rosenzweig et al., 2022) also provide excellent overviews of motivation interventions for students in K-16 contexts. Both reviews differentiated between (a) targeted interventions, which focus on shifting a specific form/aspect of motivation in order to address a specific educational problem, and (b) multicomponent (or multi-construct) interventions, which aim to change multiple forms of motivation either within a single theory (e.g., expectancies and values) or by integrating across theories in order to enhance multiple outcomes. Hulleman and Barron (2016) identified four types of targeted interventions that have been used successfully in both K-12 and university contexts: expectancy and control beliefs interventions (e.g., attributions, Perry et al., 2010; mindsets, Blackwell et al., 2007), value and interest interventions (e.g., utility value, Hulleman & Harackiewicz, 2009), goal interventions (e.g., achievement goals, Muis et al., 2013), and psychological cost interventions (e.g., affirmations, Cohen et al., 2006). They only identified two multicomponent interventions, Martin’s (2008) Motivation and Engagement Wheel and Guthrie and Wigfield’s (Guthrie et al., 2000) Concept-Oriented Reading Instruction, which were both conducted in K-12 contexts. Rosenzweig et al. (2022) took a similar approach in their discussion of interventions based in Situated Expectancy Value Theory (SEVT) and also found that there were fewer multi-construct interventions relative to targeted ones.

Focusing on targeted interventions in primarily post-secondary contexts, Harackiewicz and Priniski (2018) reviewed task value interventions, framing interventions (focused on social belonging, mindsets, and attributional retraining), and values affirmation interventions. Importantly they differentiated among course specific, field specific, and college general interventions, highlighting the variety of ways in which targeted interventions have been implemented as well as the affordances and constraints of these different approaches. Overall, they documented the benefits of task value and framing interventions both for the specific population they were designed to support as well as some broader main effects. They found less consistent evidence for values affirmation interventions, noting that this was perhaps because they were first developed for middle schoolers and thus may not function the same way in less personal college environments. Harackiewicz and Priniski also noted that some of these interventions, especially task value interventions, have investigated underlying mechanisms, with research suggesting that they may function through recursive processes, an idea that is similar to proposed processes for development within CDS approaches.

### Strengths and Limitations of Targeted Interventions

A clear strength of targeted interventions is the relative ease with which such interventions can be administered, often requiring a single or only a few sessions (as in mindset interventions; e.g. Blackwell et al., 2007; Yeager et al., 2019) or by adding reflective writing assignments to course requirements (as in utility value interventions; e.g., Harackiewicz et al., 2016). Moreover, as noted above they are also theoretically precise and focus on particular educational problems or issues, such as closing achievement gaps for underrepresented racial/ethnic minority (URM) students (see Harackiewicz & Priniski, 2018). There is clear evidence that targeted interventions are effective: for instance utility value interventions, a targeted intervention based in Eccles and Wigfield’s expectancy value theory (e.g., Eccles & Wigfield, 2020) focused on enhancing students’ sense of the usefulness of the material they are learning in different STEM fields, have reduced achievement gaps in the course in which they were implemented and show positive long-term effects on students’ performance and persistence in STEM fields (e.g., Asher et al., 2023; see also Harackiewicz & Priniski, 2018). Another clear strength is that targeted interventions often theorize about and investigate the processes by which the targeted interventions work (Harackiewicz & Priniski, 2018; see also Hecht et al., 2019; Yeager & Walton, 2011; Walton, 2014). Indeed, the ability to test these interventions in both lab and classroom contexts affords possibilities for isolating cause and effect and for identifying key mediators and moderators. Finally, targeted interventions require careful and sophisticated experimental designs, particularly when looking at long-term effects (see Harackiewicz & Priniski, 2018, for further discussion of design issues in this kind of intervention research).

Despite these clear strengths of targeted interventions, a limitation is that targeted interventions may not be beneficial for all students. For instance, Hulleman and Barron’s (2016) review of utility value interventions found that these interventions are most effective for students with low competence (perceived or actual), leading them to conclude “our recommendations are not so simple as to say that utility-value interventions are good for everyone. Instead, it is important to be mindful of individual differences when employing strategies intended to enhance motivation” (p. 167). Of course, some studies do find main effects of utility value for all students, as discussed above, and one of the points of the targeted interventions is to target particular students who may benefit from the intervention.

There is also evidence that the effectiveness of targeted interventions varies across different contexts. Yeager et al. (2019) conducted a growth-mindset intervention in a nationally representative sample of low-achieving high school students and found that the gains were greatest in schools where peer norms were to seek out challenge as well as in lower-achieving schools. These findings (and others) led Walton and Yeager (2020) to conclude that one must consider the “psychological affordances” of the context when implementing interventions such as mindset interventions, suggesting that one needs “good soil” to plant “good seeds.” Indeed, this concern about the “soil” may be especially heightened in the cases when targeted interventions focus solely on changing the person (i.e., a students’ motivation) and do not attempt to change the context. Harackiewicz and Priniski (2018) also raised important questions about when these interventions work, for whom, and under what circumstances, noting “On the one hand, the theories behind these interventions provide hypotheses about general mechanisms that should apply across contexts and populations… On the other hand, our application of theory needs to be more context dependent; we might implement the intervention differently or invoke different mechanisms for how the intervention works in particular contexts.” (p. 429). Because of this observed variation in effectiveness across contexts, researchers conducting targeted interventions increasingly are interested in considering contextual effects on their interventions, an approach that aligns with our call to investigate motivation interventions within a CDS framework.

### What About Existing Multicomponent Interventions?

Although less frequent than targeted interventions, there are a number of multicomponent interventions presented in the literature. Rosenzweig et al. (2022) identified numerous benefits of these approaches, noting that by targeting multiple forms of motivation that have been linked to positive learning outcomes, they (a) have the potential to reach more students, who may need different motivational supports, within a single classroom, (b) may interact to produce multiplicative effects on performance, (c) and may have effects on more outcomes given the varied benefits of different types of motivation on different outcomes (e.g., perceived competence with achievement; task value with choice). However, they also cautioned that there may be challenges with implementing multicomponent interventions, suggesting that they may cost more in terms of researcher time and resources, may take more time for teachers to implement, and may be less effective if they require students to engage with more materials, especially for younger students. To the extent that one is interested in doing so, it may be more difficult to determine systematically how particular aspects of a multifaceted intervention contribute to its effectiveness or to isolate the underlying psychological mechanisms or causes to explain why the intervention does or does not work (Rosenzweig et al., 2022). We return to this point later when discussing the promises and challenges of CDS approaches.

### Another Distinction: Embedded versus Add-on Approaches to Interventions

**Fig. 1:**
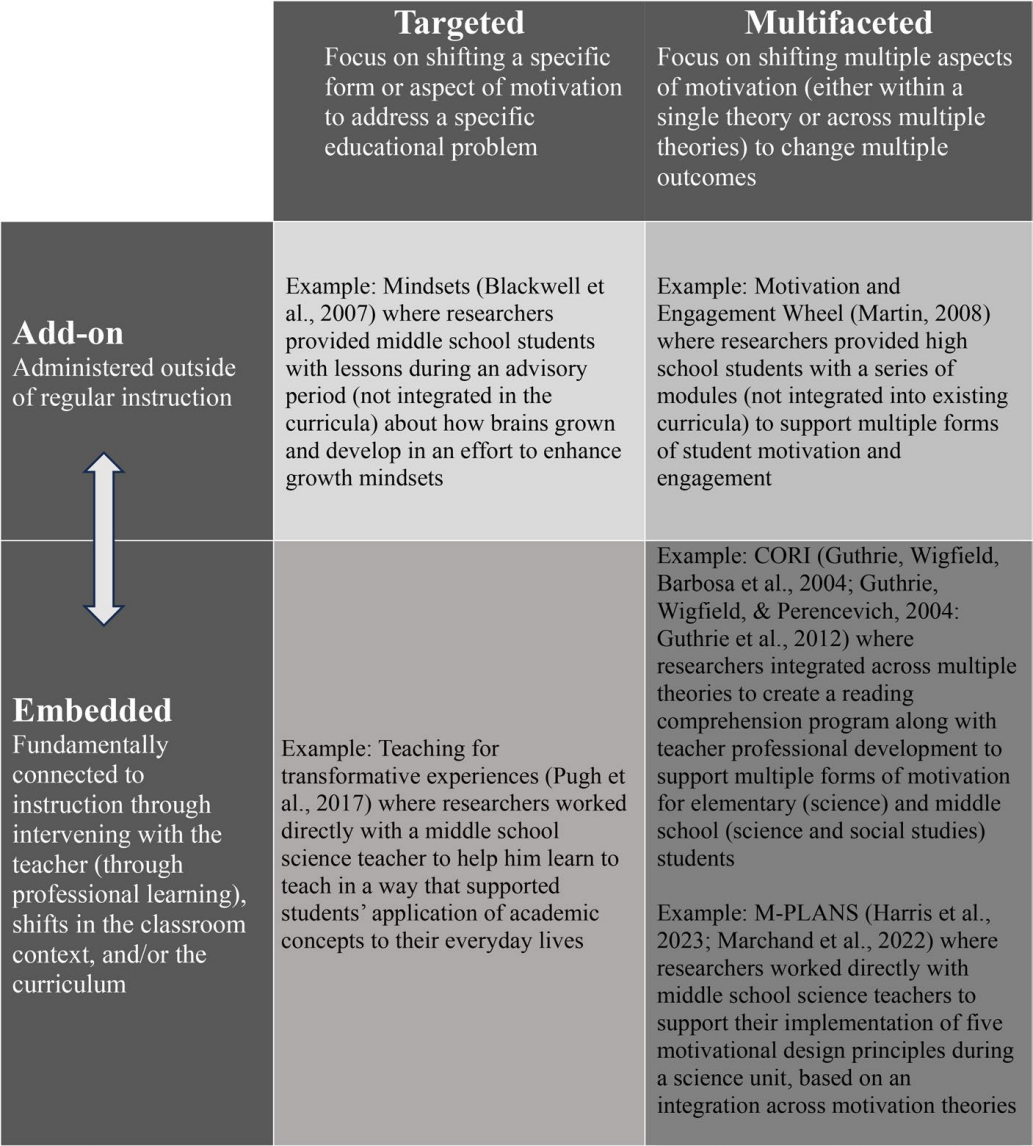
Classification system of interventions with illustrated examples. The arrow between add-on and embedded indicates that there is some continuum, such that some lessons may be fully stand-alone, others may be partially integrated into instruction, and others involve a more in-depth reframing of the curricula and/or instructional approach. Any of these four types of interventions can be framed using a Complex Dynamic Systems (CDS) framework

An important point in discussing educational interventions is that there is heterogeneity in the way that interventions are implemented, with some seeking to directly change a psychological process in a student while others seek to change the educational context (and others fall in between these two extremes). This distinction often (but not always) confounds targeted versus multifaceted approaches and thus we think it useful to more clearly separate out what the intervention is focused on (targeting one form of motivation or educational problem *versus* multiple forms of motivation or a broader array of educational problems) and the way in which the intervention is implemented (as an “add-on” module or activity *versus* embedded fundamentally in the approach to teaching, classroom context, and/or the curriculum) (see Fig. 1). Thus, in addition to “targeted” v. “multifaceted”, we also propose that researchers consider whether motivational interventions are “embedded” within the curriculum versus implemented as an “add-on” module or activity. Of course, the idea of an embedded intervention is certainly not new – much of the earlier motivation intervention research (e.g., Ames, 1992; Maehr & Midgley, 1996) was embedded. Our point in making this distinction is that we find it helpful in thinking about motivation interventions to not only differentiate between the focus (targeted v. multifaceted), as others have done (e.g., Harackiewicz & Priniski, 2018; Hulleman & Barron, 2016; Rosenzweig et al., 2022), but also the way in which it is implemented in educational settings (add-on v. embedded), which we contend operates on a continuum (as indicated by the arrow between add-on and embedded in Fig. 1).

## Where Should the Field Go Next?

We greatly appreciate the contributions of existing targeted and multifaceted interventions to the field’s understanding of how motivational beliefs and values can be changed in ways that lead to long-term positive outcomes. And, we think there is merit in extending targeted interventions to other “single” motivation constructs, such as the cost-reduction intervention work that is ongoing (e.g., Rosenzweig et al., 2020; Beymer et al., 2025). Rosenzweig et al. (2022) provided guidelines for doing targeted interventions on other constructs from situated expectancy-value theory, and the same could be done for single constructs from other theories.

However, as noted earlier, we contend that at this point in the development of the field’s knowledge, it is also time to develop more theoretically integrated, multifaceted interventions that are deeply embedded in and work to change classroom contexts in order to reach more students who face different motivational challenges and to have a more sustained impact for both current and future students. Further, we contend that such interventions should also take into consideration the complexity of students’ motivation, learning and engagement as well as the nature of the classrooms and schools that they attend. Intervening broadly in learning contexts has the potential to provide deeper impacts on students’ motivation, engagement, and learning in that students will have sustained supports for their motivation that creates a “fertile soil” for motivation to develop further. We return to this point later in our discussion of the promises and challenges of such approaches. In essence we are proposing a paradigm shift to integrate across theories to develop multiple theory, multifaceted interventions based in CDS. Next, we elaborate on these two dimensions (theory integration and CDS approaches).

### Theoretical Integration in the Design of Motivation Interventions

Several prominent theories have guided much of the work on achievement motivation conducted in the educational and developmental psychology fields over the last 40 years. These include attribution theory (Graham, 2020; Weiner, 1985), achievement goal theory (Maehr & Zusho, 2009; Urdan & Kaplan, 2020), (situated) expectancy-value theory (Eccles & Wigfield, 2020), social cognitive theory and its central construct self-efficacy (Bandura, 1997; Schunk & DiBenedetto, 2020), and self-determination theory (Ryan & Deci, 2020). We assume readers are familiar with these theories and so do not review them in detail here (see Murphy & Alexander, 2000; Wigfield & Koenka, 2020 for special issues of *Contemporary Educational Psychology* focused on these theories). The theories make fine-grained distinctions among constructs, such as self-efficacy and self-concept of ability. Interestingly, however, there is substantial overlap in the recommendations stemming from these theories regarding how educators can support the various forms or aspects of motivation included in them (Guthrie et al., 2004a, b; Jones, 2009; Linnenbrink-Garcia et al., 2016; Martin, 2008; Patall et al., 2026; Pintrich, 2003; Turner et al., 2014). Thus, for the most part, the fine-grained theoretical distinctions between related constructs do not translate into different educational recommendations (see also Anderman, 2020 for discussion of this issue).

This has led motivational scholars, especially those interested in use-inspired basic research (Stokes, 1997), to propose an integrative approach to the application of motivational theory to classroom practice. For instance, Pintrich (2003) identified 14 design principles for supporting five forms of motivation (competence-related beliefs, attribution and control beliefs, interest and intrinsic motivation, values, and goals). Building on Pintrich’s (2003) work, Martin (2007, 2008) proposed the Motivation and Engagement Wheel, which integrated across theories on motivation and engagement to propose a “wheel” consisting of 11 lower order factors grouped into four higher dimensions: adaptive cognitions (self-efficacy, mastery orientation, valuing), adaptive behaviors (persistence, planning, task management), impeding/maladaptive cognitions (anxiety, failure avoidance, uncertain control), and maladaptive behaviors (self-handicapping, disengagement). As noted earlier, Martin developed an intervention using this integrative approach, which successfully supported students’ motivation and engagement.

Jones (2009) developed the MUSIC model as a practical model to guide practitioners. It consists of five motivational climate components (eMpowerment, Usefulness, Success, Interest, Caring) and has been used to design instruction and curriculum to enhance motivation (as well as to investigate motivational climate and its connection to students’ learning and engagement), primarily in higher education (Jones et al., 2025). A recent meta-analysis of studies based on the MUSIC model found that the five components were positively related to students’ engagement, domain identification, course ratings, and achievement (Jones et al., 2025). Turner et al. (2014) also drew from multiple theories related to motivation and engagement to identify four instructional practices (supports for belonging, competence, autonomy, and meaningfulness) that they argued would support a variety of forms of motivation; they found that teachers trained in these four instructional practices shifted their instruction, with resulting changes in students’ engagement. Most recently, Urhahne and Wijnia (2023) integrated across expectancy-value theory, social cognitive theory, self-determination theory, interest theory, achievement goal theory, and attribution theory to create a framework based on Heckhausen and Heckhausen’s (2018) action model, highlighting how constructs from these major theories work together to explain students’ learning.

The two interventions, that we highlight below, were built on integrative perspectives. CORI (e.g., Guthrie et al., 2004a, b) was developed by integrating across multiple motivational theories including goal theory, interest theory, self-determination theory, expectancy-value theory, and social cognitive theory, and cooperative learning as well as theory and research on effective reading strategies to develop a motivationally supportive reading intervention for both elementary and middle school students. M-PLANS (Harris et al., 2023; Marchand et al., 2022) was based on Linnenbrink-Garcia et al.’s (2016, see Patall et al., 2026 for an expanded discussion) five motivational design principles, which were developed by integrating across motivation theories to identify contextual supports for competence beliefs, effort attributions and growth mindsets, value and interest, intrinsic motivation, mastery goal orientations, and positive emotions. We describe both theoretical integrations and their translation to instructional practices in greater detail below.

More broadly and in the spirit of this special issue, we believe theoretical integration is needed because at this point of the field’s development no theory captures all relevant motivation constructs that impact student learning outcomes. Perhaps no one theory ever can; we agree with Graham (2020) who stated, following Kelly (1955), that theories have foci and a range of convenience, meaning they cannot encompass all of motivation because it is too complex. But given both the overlap in constructs just discussed as well as the unique constructs present in different theories, we contend that more fully integrative approaches are needed so that motivation interventions impact more students.

Although we are advocating for integration, we agree with Pekrun (2024) and Ryan (2024) who say that theoretical integrations should be done carefully and conceptually. Pekrun provided a set of rules for deciding when theories could or should be integrated; we note two of them here. First, if theories are studying the same phenomena, then that is a good reason to integrate them. Second, if theories make converging predictions regarding motivation and outcomes, then merging may be possible. Ryan (2024) urged researchers to approach integration in a systemic fashion. He stated that just adding constructs to a given theory without considering carefully how they fit into the theory does not advance the field. He also stated that researchers interested in integrating theories should specify how doing so leads to new insights and provide a better explanation of the phenomena that the individual theories did. Thus, we encourage readers to think carefully about how and why they are integrating across theories before doing so.

### A CDS Approach to Motivation and Human Functioning

Beyond theory integration, we also contend that there is another way for motivational researchers to look beyond current dominant approaches to motivation intervention. Specifically, considering motivation as part of a complex dynamic system (CDS) may yield additional insights into how to create motivation interventions that support and sustain students’ motivation and engagement (see also Kaplan, 2023 on this point).

Interest in understanding education as a complex dynamic system is growing (e.g., Davis & Sumara, 2006; Hilpert & Marchand, 2018; Kaplan & Garner, 2020; Koopmans, 2020; Marchand & Hilpert, 2020, 2023). CDS served as an underlying framework for the M-PLANS motivation intervention, described below, and has also been embraced by other motivational scholars (e.g., Kaplan & Garner, 2017; Skinner et al., 2022; Wilkins et al., 2025). Below, we present several key aspects of CDS as a brief primer for scholars who may be less familiar with this approach (see Davis & Sumara, 2006; Hilpert & Marchand, 2018; Kaplan & Garner, 2020; Koopmans, 2020; Marchand & Hilpert, 2020, 2023 for more thorough discussions).

A CDS approach is based on the idea that humans’ psychological processes and characteristics can be characterized as organized systems embedded in complex environments that are themselves part of layered and complex systems (Magnusson & Cairns, 1996; Marchand & Hilpert, 2023). These systems are dynamic, meaning that they are self-organizing, adaptive, and emergent (Smith & Thelen, 2003; Thelan, 2005). The interactions amongst the elements within the system generate novel patterns of behavior. The way elements combine is dependent on other interactions amongst systems elements, therefore behavior is multicausal and reflects patterns of instability and stability over time within the system. Complex systems also consist of multiple time scales, ranging from millisecond to years, so that behavior may look different depending on when it is observed and for what length of time.

Another key idea of CDS is that change is non-linear: behavior changes in different ways due to different experiences (Koopmans, 2020). Change also can happen dramatically (rather than slowly) when a small event causes a large change throughout the system, referred to as developmental cascades. Further, there are certain patterns of psychological processing or behavior that students gravitate towards; these are called attractor states, and they can stabilize behavior but are also more difficult to change. For instance, one could imagine that it might be challenging to move a student who is oriented to outperforming others (performance goal orientation) and views intelligence as fixed to focus on developing their competence (mastery goal orientation) and viewing intelligence as malleable. Thus, a goal of an intervention would be to try to shift and change the patterns in the extant attractor states to more positive ones. Heino et al. (2023) proposed that one could visualize attractor states as a landscape, where attractor states are represented by valleys. In this analogy, deeper valleys represent stronger attractor states. If one were to represent an individual with deeply held and consistent behavioral patterns as a ball on the landscape, they would be more likely to move towards the deeper valleys, and it would be harder to move them out of a deep valley. With this metaphor, intervention can be thought of as helping to shift and change the attractor state landscape such that they can deepen valleys (create stronger attractor states for “adaptive” forms of motivation) or help to flatten the landscape where the existing “deep valleys” represent recurrent forms of less adaptive motivation or engagement that the intervention is trying change, thus facilitating shifts in beliefs or behaviors away from the less desired attractor states.

Until recently, empirical research based in the major motivation theories mentioned above often ignored this notion of dynamic complexity, focusing instead on linear models and attempts to identify causal relations between constructs. Despite this methodological approach to research, we contend that the idea that motivation is itself a complex system embedded within broader complex systems is compatible with many modern motivational theories. First, the theories are based on the proposition that students’ motivation is formed, at least in part, through socialization processes, such as interactions with their parents, teachers, and peers and that these socialization processes are embedded within the larger socio-historical context (e.g., Bandura, 1997; Maehr & Zusho, 2009; Eccles & Wigfield, 2020; Renninger & Hidi, 2016; Ryan & Deci, 2020; Skinner et al., 2022). In this way, motivation theorists acknowledge the idea of a multi-layered system. For the purpose of developing motivation interventions, an important implication of this notion of nested systems of influence is that aspects of the classroom context that can be changed via intervention, such as through changes in curricula or professional development focused on shifting teachers’ instructional practices, can be introduced into the system and potentially play an important role in shaping students’ motivation, engagement, and learning within that context. In this way, one could think of motivation interventions as helping to shift the attractor state landscape and thus push and pull students towards or away from certain forms of motivation.

Second, existing theories do not view motivation as static or stable but rather posit that it can shift and change based on both the context as well as other forms of motivation, typically in ways that cannot be reduced to a single cause. There are numerous longitudinal studies providing clear evidence of changes in motivation across the K-16 context (e.g., Gaspard et al., 2017; Jacobs et al., 2002; Middleton et al., 2004; Musu-Gillette et al., 2015; Robinson et al., 2018). Research also indicates that there are shifts and variations in motivation over shorter time spans, such as a classroom unit or single semester (e.g., Palmer, 2009; Kosovich et al., 2017; Martin et al., 2015). While the majority of this research is based on linear change models, there is emerging evidence of the dynamic nature of change (e.g., Benden & Lauermann, 2022; Dietrich et al., 2017; Parrisius et al., 2022; Wolff et al., 2024). For instance, in a study of college mathematics students, Benden and Lauermann (2022) observed a rapid shift in task values and perceived costs in relation to performance feedback on math worksheets in competence beliefs in relation to exam feedback. This potential dynamism of motivation is important because it implies that students’ motivational beliefs emerge through a complex interplay of the individual embedded within contextual systems. As such, changes in the educational context can contribute to the emergence of different patterns and systems of motivation, engagement, and learning. For instance, Geerling et al. (2020) applied a CDS framework to examine how patterns of confusion and interest changed over time in response to a utility value intervention.

Third, the various forms of motivation are closely connected to each other in extant theories. Earlier research on motivational processes has focused on linear relations, such that a change in one form of motivation is associated with a consistent degree of change in another form. For instance, competence-related beliefs are closely connected to subjective task values such that students who are confident in their abilities in a domain often hold higher value beliefs in that same domain (Jacobs et al., 2002; Mac Iver et al., 1991). Moreover, the strength of the relation between competence beliefs and subjective task values increases across development (see Wigfield, 1994). Researchers now have extended this work by proposing that these competence beliefs, task values, and other aspects of motivation are part of emergent systems whereby beliefs systems are reinforcing and may become more integrated (and potentially stable) over time (see Barger & Linnenbrink-Garcia, 2017; Dweck, 2017; Renninger & Hidi, 2016). Indeed, researchers have identified multiple ways motivation combines yielding different patterns of engagement and learning (Braten & Olaussen, 2005; Conley, 2012; Shell & Husman, 2008; Tamura et al., 2022), an idea that is also growing in other areas of study such as self-regulated learning (e.g., Saqr & López-Pernas, 2024).

In summary, we contend that students’ motivation should be conceptualized as a complex dynamic system. Moreover, we further contend that the field would benefit from more researchers attempting to design and implement theoretically integrated, multifaceted motivation interventions that situate motivation as part of a complex dynamic system (see Kaplan, 2023 for a similar point). It is beyond the scope of the current manuscript to develop a complete integrated model of motivation based on this general premise, but such a model could include the well-established beliefs, values, goals, and needs that are commonly studied by modern motivational scholars as part of a complex dynamic system.

As an aside, we acknowledge that it is certainly possible for motivation interventions that target a single aspect of motivation to shift a complex system (see for instance Geerling et al., 2020 discussed above). Additionally, prior arguments for brief psychological interventions in education are based on the premise that a relatively simple intervention can instigate change in the system of thought processes in which students engage when they are doing their assignments and attending class (see Hecht et al., 2019; Yeager & Walton, 2011). And, as noted earlier, intervention researchers doing targeted interventions increasingly are attending to contextual effects that can impact the results (see Harackiewicz & Priniski, 2018). In this way, it is certainly possible for targeted interventions to align with the CDS framework we suggest here. However, most targeted interventions are not designed to extensively change the educational contexts that students are in and so do not alter the landscape of potential attractor states, as discussed by Heino et al. (2023), or create the fertile “soil” for motivation to develop further (Walton & Yeager, 2020).

Moreover, we propose that multifaceted interventions designed to support multiple forms of motivation that are beneficial for learning and engagement are likely to meet the motivational challenges of more students within a single classroom than those that only target a single aspect of motivation. As noted earlier, students who already see, for example, the usefulness of what they are learning will not likely benefit from a utility value intervention - but they may be experiencing other motivational challenges that a multifaceted intervention can address.

To illustrate what multifaceted interventions look like in practice, we next present two examples of theoretically integrated and embedded motivational interventions, one of which, the M-PLANS program, reflects a CDS approach. These interventions were conducted at the elementary and middle school levels. We present them in some detail so that researchers interested in developing such interventions in the future have models for doing so.

## Concept Oriented Reading Instruction (CORI)

As noted above, CORI is a reading comprehension instructional program that focuses on enhancing not only reading comprehension through reading strategy instruction but also students’ motivation and engagement in reading. Prior to CORI most of the work in the reading field had been on the cognitive aspects of reading, and so there was scant research focused on motivation for reading, and even less work on how students’ reading motivation might be enhanced in classrooms. The strategy instruction component consisted of teaching students six different reading strategies documented in the National Reading Panel Report as being effective for increasing students’ reading comprehension. The novel part of CORI was the motivation enhancement component, which consisted of teaching practices designed to enhance different aspects of students’ motivation for reading, and more broadly, their engagement in reading (which includes their strategy use). We focus here on the motivation enhancement practices, for information on the strategy instruction component see Guthrie et al. ([Bibr CR41][Bibr CR42], 2012). We begin by discussing the theoretical grounding, approach to professional development, and implementation of CORI at elementary levels. Next, we highlight key changes in CORI for the middle school level and then provide an integrated discussion of the main findings from CORI at both levels.

### CORI at the Elementary Level

#### Motivation Enhancing Practices and Their Theoretical Bases

The various motivation enhancing practices used in CORI were grounded in several motivation theories. The right side of Table 1 provides an overview of the motivational and strategy instructional practices used in the elementary-version of CORI.

**Table 1:** Motivational and strategy instructional practices for elementary and middle school implementations of CORI

Elementary School	Middle School
**Motivational Instructional Practices**	
Thematic Unit/Knowledge Content Goals	Thematic Unit/Knowledge Content Goals
Hands-on Experiences Related to Text	Importance of Science Reading
Supporting Student Autonomy	Relevance of Science Reading
Collaboration Support	Supporting Student Autonomy
	Collaboration Support
	Experiencing Success
**Strategy Instructional Practices**	
Activating Background Knowledge	Inferencing
Questioning	Summarizing
Searching for Information	Concept Mapping
Summarizing	
Organizing Graphically	
Structuring Stories	

The first practice focused on *providing knowledge content goals/identifying the thematic unit*, which refers to identifying core learning goals for particular topic areas that provide students with compelling cognitive reasons for learning the material. These practices are based broadly in goal theory as espoused by personality and social psychologists (e.g., Fishbach & Ferguson, 2007; Pervin, 2015). When students know the goals behind what they are learning, they are more likely to embrace the material and understand why they are learning it.

*Hands on activities related to the reading activities* are connections between the academic curriculum and the personal experiences of the learners, and more specifically, are stimulating activities that connect students to the content they are learning. These practices are based in interest theories (e.g., Hidi & Renninger, 2006), which delineate phases of interest development and their relations to learning. *Interesting texts* were provided in the CORI classrooms to supplement the classroom and school library’s reading materials. These were all trade books and were at different reading levels so that all students could find books they could comprehend (see Davis & Tonks, 2004, for discussion of the selection process for these books).

*Supporting student autonomy* means giving students some control over their learning, by providing them with choices and allowing them to pursue their own questions. This practice is based in Ryan and Deci’s (2017, 2020) self-determination theory in which they propose that autonomy is a fundamental human need. One of the key things the CORI team learned in developing ways that teachers could support students’ autonomy was that the choices had to be contextualized within the classroom situation in which they were occurring, and constrained to choices among relevant reading materials, rather than being completely open (see also Patall, 2020).

*Collaboration support* refers to teachers’ encouragement of students’ interactions with other students about what they are reading. This helps them understand various perspectives about what they are reading and ties to children’s increasing interest in socializing with their peers. These practices are based in providing cooperative learning (e.g., Slavin et al., 2003) and social motivation (e.g., Patrick et al., 2011).

#### Teacher Professional Development

There are different approaches to teacher professional development, ranging from brief presentations of whatever program the researcher wants to implement to providing teachers with fully scripted programs to implement. Success for All (Slavin & Madden, 2010) is an example of a fully scripted program. CORI is somewhere in between those two poles both with respect to how the professional development was done and the kinds of materials presented in it. Over the course of the various CORI projects, the developers changed (sometimes dramatically so) the ways they were doing PD.

At the elementary school level, CORI teachers participated in a 10-day workshop during the summer to learn how to teach the various reading strategies and also learn both about different aspects of students’ motivation and then instructional practices to foster it. Teachers also designed science activities to use in their instruction and helped identify books that they could use in their classrooms. It is important to note that the research team worked to ensure that both the science topics and activities as well as the reading instructional practices aligned closely with the school district’s goals for each subject area; doing so was a key reason the district supported the project.

Additionally, a second group of teachers participated in a Strategy Instruction (SI) only component (see Table 1). The SI teachers participated in a five-day workshop that consisted of the same information provided to the CORI teachers about how to teach the reading strategies. SI teachers also learned about reading self-efficacy and ways to enhance it; however, the research team did not present anything about any of the other motivational instructional practices nor about the science activities that were part of CORI.

As part of the PD, the researchers developed comprehensive teachers’ guides for both CORI and SI that provided specific examples of how to teach a given reading strategy or how to impact the different aspects of students’ motivation. They were organized day by day for each week of the program. Each day included different reading activities (e.g., promoting fluency and vocabulary, guided reading comprehension instruction, independent reading), and implementation of activities to promote motivation (e.g., observations, hands-on activities, generating questions, etc.).

The team designed the guides to be modifiable by teachers if they wished to do so or to be used in their entirety if that is what teachers wanted to do. They did so in response to different teachers’ reactions to the workshop; some were quite comfortable elaborating and extending what was in the guides, while others wanted very specific guidance. Initially the team took a relatively top-down approach to the PD days. For instance, members of the team gave talks/lectures on the different reading strategies that were part of both CORI and SI and also on motivation. However, the team realized that teachers were not resonating to this approach, so the team changed it to a more bottom-up approach. The end result for the CORI PD sessions was an approach that was similar to how the M-PLANS team approached PD, as discussed below.

#### Implementing CORI

CORI-elementary school was a 12-week instructional program that paired science and reading as a way to provide content goals for instruction. Each class session lasted about one hour, with a total of 60 instructional hours. The science part of CORI focused on nine core biological constructs dealing with animal survival. In the first six weeks, the cognitive reading strategies shown in Table 1 were introduced sequentially over the course of a week’s reading instruction. In the second six weeks, the strategies were presented in pairs rather than individually. The motivation enhancing practices also were introduced singly in the first six weeks and then paired up in weeks seven to 12.

The teachers in the SI condition introduced the reading strategies one at a time for the first six weeks and then paired strategies for the second six weeks; they did this in ways that were quite similar to how the strategies were introduced in CORI. There were no explicit motivation enhancing practices included in the SI condition, no interesting trade books provided, and no direct connection to a subject matter. Along with the SI condition, a second study also included classes using the district’s regular reading program as another comparison group or condition.

### CORI at the Middle School Level

The research team revised the content of CORI relatively extensively for the middle school project. Two versions were created, one for science and one for social studies. The motivation enhancing strategies were changed to a degree in response to middle school students’ motivational issues and what middle school students told the team about middle school science–that it was boring, irrelevant, and hard (see Guthrie et al., 2013). In addition, the cooperating school district allotted a shorter amount of time for the CORI intervention than was available in the elementary school project.

#### Motivation Enhancing Practices and Their Theoretical Bases

The middle school version of CORI utilized many of the same motivational instructional practices as the elementary version (see Table 1), with some modifications, as discussed below. At middle school, CORI continued to be embedded in *thematic units*. CORI continued to emphasize *providing autonomy* support and *opportunities for collaboration* around reading. The CORI team increased the practices designed to enhance students’ task value based on results of an interview study with a group of 7th grade students in the school district, who said that their science reading materials were difficult, boring, and irrelevant to them (see Guthrie et al., 2012 for discussion of this study). Specifically, the team highlighted *relevanc*e, which involves helping students see that what they are learning has implications for their own lives, and *reading importance*, which helps students understand why it is important to be a good reader. These both are based in Eccles and Wigfield’s expectancy-value theory (Eccles & Wigfield, 2024), who stated that students’ valuing of a subject was determined by how interested they are in it, how important it is and whether it ties to one’s sense of self, and how useful the task is to the student. The CORI team also focused on *experiencing success*, which is how teachers work to provide success opportunities for all students in order to build their confidence in their reading skills. Helping students at all levels of achievement experience success during reading is based both in Bandura’s (1986) social cognitive theory and its key construct of self-efficacy as well as expectancy-value theory (Eccles & Wigfield, 2020), with its related constructs of expectancies for success and self-concept of ability being key beliefs that impact children’s persistence and performance. More details on these practices and how they are implemented are provided in Guthrie et al. (2004a, b).

#### Teacher Professional Development

At middle school, the CORI team took a much more bottom-up than top-down approach to the middle school PD. The PD also was much shorter, lasting for three half-day sessions. The team started the session by having teachers engage in a “mini-CORI” experience that involved a science activity. The CORI PD team introduced the various motivation enhancing practices in this session. As in the elementary school version, project staff developed comprehensive guides for each version of CORI. At both elementary and middle school levels, once teachers started implementing CORI or SI in their classrooms, project staff provided help and guidance when needed.

#### Implementing CORI

There were two versions of CORI that were implemented in middle school. The first also focused on science (see Guthrie et al., 2013 for complete description) and lasted 6 weeks. The second focused on history and social studies rather than science; the particular topic was the Civil War and lasted four weeks (see Guthrie & Klauda, 2014).

### CORI’s Impact on Students’ Reading Motivation and Reading Achievement: Key Results from the Elementary and Middle School Studies

Guthrie et al. (2004a, b) reported results of two quasi-experimental matched group design studies that tested the effectiveness of CORI vs. SI (Study 1) and vs. SI and Traditional Instruction (TI, Study 2). Results from Study 1 indicated that CORI students reported higher reading motivation, made greater use of the reading strategies, and had higher reading comprehension (on project-created measures) relative to SI students. In Study 2, CORI students again had higher reading motivation, and outperformed SI and TI students on the Gates-MacGinitie Reading Tests (effect sizes of 1.40 and 0.71, respectively); few reading intervention studies have produced differences in standardized achievement test scores between treatment and control groups, speaking to the benefits of combining motivation and reading strategies - teachers rated their students as higher in reading self-efficacy, intrinsic motivation in reading, and extrinsic motivation in reading than did the SI teachers (effect sizes 0.95, 1.29, and 1.28, respectively). These effect sizes are substantially higher than the average effect sizes for motivation interventions reported by Lazowski and Hulleman (2016) in their review of targeted and multifaceted motivation intervention work.

How can these results be understood and explained? The only systematic difference in how the CORI and SI conditions were implemented was the motivational instructional practices; thus, one possible conclusion is that the addition of the motivation practices explained the differences observed across condition. However, Guthrie et al. (2004a, b) also proposed that the motivationally supportive practices and the reading strategy practices likely interacted in complex ways to influence students’ comprehension and motivation. In this sense, they were anticipating some of the ideas put forward by CDS theorists regarding the complex interplay of instruction, student, and content in classrooms.

Building on this work, Guthrie et al. (2007) conducted a meta-analysis of CORI’s effects on student outcomes. They summarized 11 CORI studies involving elementary and middle school students, with each study containing CORI groups and comparison groups. The effect sizes (*d’s)* indicate the strength of the differences in posttest scores on the different measures between CORI and comparison groups. The mean effect sizes across the studies for students’ self-reported intrinsic motivation, self-efficacy, teacher ratings of student engagement, students’ standardized reading comprehension test performance, performance on a researcher-generated reading comprehension test, and students’ reading strategy use (actual strategy use, not self-reports) are shown in Table 2. They range from a low of 0.49 for students’ self-efficacy to 1.20 for the intrinsic motivation composite. As can be seen, nearly all of the effect sizes are larger than those Lazowski and Hulleman (2016) reported in their summary of motivation interventions.

**Table 2:** Average effect sizes of motivation, engagement, reading comprehension, and reading strategy use across 11 CORI studies

Variable	Mean Effect Size (*d*)
Intrinsic Motivation Composite	1.20
Student Self-Efficacy for Reading	0.49
Teacher Ratings of Student Engagement in Reading	1.00
Standardized Reading Test Comprehension	0.91
Multiple Text Comprehension	0.93
Student Reading Strategy Use	0.91

Meta-analytic data is from Guthrie et al. (2007)

For the middle school intervention, we focus here on results from the history/social studies version of CORI at middle school, which used a very powerful switching replications design in which students experienced both the treatment and control group experiences, and as a result acted as their own controls (see Guthrie et al., 2013; Guthrie & Klauda, 2014, for discussion of this design). In the first phase of the study, some students received CORI and others received the district’s traditional instruction (the control group). The groups switched after four weeks so that the treatment group became the control group, and the control group received the treatment. This design is a powerful one that has high internal validity. Each student experienced both the treatment and control instruction; thus, much within-individual variance is controlled for and so is not a concern when interpreting the results (Trochim, 2005). Two major findings from the study were that students in the CORI condition increased in their reading comprehension (either when they were in CORI first or second) and students in the TI group did not. Second, increases in motivation and engagement were more strongly associated with the motivation enhancing instructional practices in CORI than those in TI.

In summary, Guthrie and Wigfield demonstrated that CORI boosted both elementary and middle school students’ reading comprehension, actual reading strategy use, motivation, and engagement in reading. In their discussion of the elementary school results, Guthrie et al. (2004a, b) stated that “Although it may be possible to isolate the effects of these different instructional practices in experimental studies, we believe that in classroom settings, these practices depend on each other to some degree” (p. 417). Results of CORI show the benefits of taking an integrative approach to interventions done in classrooms and so provide support to our overall thesis that such approaches are important for enhancing more students’ motivation in “real” classroom settings.

## Motivation-Planning Lesson to Activate eNgagement in Science (M-PLANS)

M-PLANS drew from Linnenbrink-Garcia et al.’s (2016) articulation of a set of motivational design principles (MDPs) based on multiple motivational theories. The idea was that teachers could draw upon these MDPs to support students’ motivation, engagement, and learning in ambitious science instruction at the middle school level. The design principles and the overall implementation of these MDPs, discussed in greater detail in the section that follows, build upon and extend the approach used in CORI for supporting student motivation (indeed, CORI was part of the inspiration for the M-PLANS project).

### Motivation Enhancing Practices in M-PLANS and Their Theoretical Bases

The starting point for the M-PLANS professional learning (PL) approach was Linnenbrink-Garcia et al.’s (2016) five design principles for supporting motivation and emotion in classroom contexts: (1) support competence through well-designed instruction, challenging work, and informational and encouraging feedback, (2) support students’ autonomy through opportunities for student decision making and direction, (3) select personally relevant, interesting activities that provide opportunities for identification and active involvement, (4) emphasize learning and understanding and de-emphasize grades, competition, and social comparison, and (5) support feelings of relatedness and belonging among students and with teachers. As discussed earlier, these design principles were developed based on extensive prior research on both the types of motivation that support students’ engagement, learning, and decisions to pursue science careers as well as the contextual factors that help to support the development (and maintenance) of these beliefs.

Building from these five integrative design principles, the M-PLANS team engaged in an extensive co-design process whereby motivation and science education researchers collaborated with in-service middle school science teachers as well as district and county science coordinators to refine the design principles to be specific to science (see Table 3) and create a professional learning approach that helped middle school science teachers enact these design principles in their middle school science classrooms. One key goal of this work was to align the design principles with the Next Generation Science Standards (NGSS: NGSS Lead States, 2013) so that the recommendations and strategies for enacting the MDPs supported the type of learning called for by the *Framework for K-12 Science Education* (National Research Council, 2012). In this way, the M-PLANS team took the domain-general design principles identified by Linnenbrink-Garcia et al. (2016) and situated them within middle school science. We describe each design principle below, highlighting its theoretical basis as well as how its enactment.^2^

**Table 3:** Motivational design principles that support (or reduce) various motivational beliefs

Motivational Design Principles		Motivational Belief Supported	Theoretical Basis
Belonging	Support feelings of relatedness and belonging within the classroom community and the larger science community by promoting inclusion and helping all students to identify with science	Supports task values (attainment, utility, interest), intrinsic motivation; reduces extrinsic motivation	Self Determination Theory (Ryan & Deci, 2017); Belongingness Motivation (Baumeister & Leary, 1995)
Confidence	Support students’ confidence for science through instruction that includes clear expectations; challenging work that is calibrated to the knowledge, skills, and abilities of students; and informational and encouraging feedback	Supports competence beliefs, mastery goal orientations, intrinsic motivation	Social Cognitive Theory (Bandura, 1997); Situated Expectancy-Value Theory (Eccles & Wigfield, 2020); Self Determination Theory (Ryan & Deci, 2017); Attribution Theory (Weiner, 2010)
Learning Orientation	Emphasize growth in three-dimensional learning and understanding as the goal of science learning, rather than rote learning, grades, competition, or social comparison	Supports competence beliefs, mastery goal orientations; reduces performance goal orientations, extrinsic motivation	Achievement Goal Theory (Ames, 1992); Theories of Intelligence (Dweck, 1999)
Autonomy	Support students’ autonomy through opportunities for student decision making and direction during science instruction, such as in the context of investigations of phenomena and solving problems	Supports task values (attainment, utility, interest), intrinsic motivation, mastery goal orientations, competence beliefs; reduces extrinsic motivation	Self-Determination Theory (Ryan & Deci, 2017); Stage-Environment Fit (Eccles et al., 1993)
Relevance	Provide opportunities for learning science that students find personally meaningful, interesting, and/or culturally relevant	Supports task values (attainment, utility, interest), mastery goal orientations	Situated Expectancy Value Theory (Eccles & Wigfield, 2020); Interest Theory (Hidi & Renninger, 2006); Culturally Relevant/Responsive Education (Ladson-Billings, 1995)

The first principle, *Belonging*, calls for supporting feelings of relatedness and belonging within the classroom community, the domain of science, and the community of practicing scientists by promoting inclusion and helping all students identify with science. Multiple motivation theories are built on the assumption that individuals have a need to be related to others (Baumeister & Leary, 1995; Ryan & Deci, 2017). Studies with students from elementary school through college show positive associations between relatedness to teachers and peers with a variety of desirable academic outcomes including intrinsic motivation, grades, self-efficacy, and engagement (Beachboard et al., 2011; Furrer & Skinner, 2003). Feeling a sense of belonging to science and a connection (or potential connection) with the scientific community is also important for learning and identity development (Carlone & Johnson, 2007).

The *Confidence* MDP suggests supporting students’ self-competence beliefs, such as self-efficacy, expectancies, and academic self-concept, through instruction that includes clear expectations, challenging work that is calibrated to the knowledge, skills, and abilities of students, and informational and encouraging feedback. Students’ positive self-assessments of their ability and likelihood of succeeding at a task are highlighted as key motivational beliefs by multiple motivational theories (Bandura, 1986; Eccles & Wigfield, 2020; Ryan & Deci, 2017; Weiner, 2010). Prior research shows that students’ perceived competence is predictive of a variety of academic outcomes including academic achievement, intrinsic motivation, effective strategy use, and academic satisfaction (Usher, 2016). Of note, this MDP was originally named “competence” but was changed to “confidence” based on co-design work with teachers who often associated the notion of competence with students’ actual skill development rather than students’ beliefs about their ability to learn and succeed in science.

The third principle, *Learning Orientation*, emphasizes growth in three-dimensional learning and understanding as the goal of science learning, rather than a focus on rote learning, grades, competition, or social comparison. Three-dimensional learning conceptualizes learning in science in relation to (1) scientific practices for investigation and model building, (2) concepts that cut across domains of sciences (e.g., identifying patterns), and (3) disciplinary core ideas related to the physical sciences, life sciences, earth and space sciences, and engineering and technology (National Research Council, 2012). Several related motivation theories assert that students benefit when an emphasis is placed on developing competence and understanding rather than demonstrating performance (see Ames, 1992; Dweck, 1999; Weiner, 2010). A learning orientation contrasts with a performance orientation, which is focused around demonstrating one’s competence (often in the form of achieving high grades or outperforming others) and avoiding failure. Students who adopt a learning orientation demonstrate higher academic engagement and sometimes perform better academically (Urdan & Kaplan, 2020).

Fourth, the *Autonomy* MDP suggests supporting students’ autonomy through opportunities for student decision making and direction during science instruction, such as in the context of investigations of phenomena and solving problems. Providing support for autonomy also involves providing rationales and support rather than using controlling language or actions and taking steps to acknowledge student perspectives. Theory suggests that teacher support for autonomy is critical for supporting students’ motivation and engagement (Eccles et al., 1983; Ryan & Deci, 2017) and has been linked to students’ engagement, self-regulation, self-esteem, and academic achievement (Assor et al., 2002; Jang et al., 2016; Soenens & Vansteenkiste, 2005).

The final MDP, *Relevance*, asks teachers to consider students’ perspectives and provide science learning opportunities that students find personally meaningful, interesting, and/or culturally relevant. For instance, teachers might use project-based learning or personally meaningful activities that draw students in or help students connect what they are learning to their daily lives or future goals (Blumenfeld et al., 2006; Harris & Allen, 2022). This MDP is derived from both Interest Theory (Hidi & Renninger, 2006) and Situated Expectancy Value Theory (Eccles & Wigfield, 2020). As teachers enact this MDP, they should consider relevance in relation to the population of students they teach rather than relevance as reflected in only European American cultural knowledge and standard curricula (Ladson-Billings, 1995).

### Development and Implementation

As described in Marchand et al. (2022), M-PLANS was co-developed using a design-based research approach^3^ (Design Based Research Collective, 2003), a framework developed to carry out formative research on educational designs with the explicit aim of refining those designs through multiple iterations (Collins et al., 2004). The focus on learning processes and development of situated theory through iterative processes of intervention and revision make design-based approaches highly relevant to the development of instructional strategies. Using this approach, the M-PLANS team was able to consider the emerging instructional needs for supporting students’ motivation situated within the context of more demanding science instruction. Additionally, the approach to professional learning was developed to reflect four themes from contemporary theories of adult learning: (a) experience is foundational to adult learning; (b) adult learning is reflective, often involving planning, monitoring, and reflecting on experiences; (c) adult learners benefit from dialogue aimed at developing common understanding and support; and (d) adult learning is shaped in important ways by context (Rohlwing & Spelman, 2014). Following Guskey’s (2014) reverse design approach, the M-PLANS team engaged teachers in an interactive process of collaboration, enactment, and reflection (Krajcik et al., 1994) around the design of their lessons and instructional strategies that are informed by and appropriate for their unique school and classroom contexts. Comparing the M-PLANS approach to professional learning to that of CORI, the M-PLANS team essentially started with the approach with which CORI ended.

The co-design project occurred in three broad phases. In Phase 1, the research team began to develop and design a PL curriculum, with feedback from both science teachers and district-level science coordinators about their views on motivation in the context of middle school science. In Phase 2, the team implemented, tested, and began to refine the PL approach in collaboration with six co-design teachers whose classrooms served as sites for the first round of data collection. These teachers participated in a 3-day summer PL program where they learned about the five MDPs. As part of the co-design process, they also provided feedback on the PL as well as other tools developed to support MDP-aligned instruction (e.g., end-of-class (ECR) student and teacher surveys to help gauge students’ motivation and engagement; a lesson-planning tool to support teachers’ incorporation of motivational practices into NGSS-aligned lessons). Teachers then enacted the MDPs across their seventh grade NGSS-aligned science units focused on chemistry (the curriculum materials varied across schools, but all curricula were based on the same NGSS performance expectations), with half of the co-design teachers enacting first while receiving frequent feedback from the research team and the three co-design teachers who were not implementing and then the other three teachers followed the same process. After both units were complete, the researchers, co-design teachers, and science coordinators met to refine the M-PLANS PL program. Extensive quantitative and qualitative data were collected from both teachers and students before, during, and after implementation.

As in the CORI PD process, the refinements resulted in significant changes to M-PLANS PL. For instance, the team removed a complex and burdensome lesson planning rubric aimed to help teachers evaluate whether their lessons aligned with the MDPs and instead incorporated guiding reflection questions for each MDP for teachers to help them consider specific strategies they could use to enact the MDP in the upcoming lesson. The team also developed a comprehensive toolkit (available on m-plans.org) that included more concrete examples to illustrate the MDPs, helped to show the connection to NGSS, and provided guidance on how to support equitable science instruction as well as general classroom organization, routines, policies, and climate considerations in relation to the MDPs. A set of “quick tools” that included pocket-sized flipbooks and activity cards designed for teachers to use as just-in-time supports while planning and teaching were also developed. As discussed by Marchand et al. (2022), these extensive revisions reflected a need to (a) move from more abstract principles to concrete suggestions, (b) address the broader classroom climate, and (c) provide stronger supports for teacher reflection.

Phase 3 involved a broader implementation and testing of the M-PLANS PL. In the summer before the academic year, 18 teachers participated in a refined 3-day PL where they learned about the MDPs and accompanying resources and practiced implementing them. These teachers then enacted the MDPs during their chemistry-focused science unit. To be clear, the goal of the PL was to help teachers use their professional judgment about when and how to implement the MDPs in respect to their specific classes and lessons. As such, the intervention focused on changing teachers’ beliefs and knowledge while the PL provided the tools they needed to shift their practice. The idea was that these shifts in practice would adjust the motivational system landscape (i.e., to deepen the valleys of positive forms of motivation and engagement and flatten the less adaptive forms of motivation and more superficial engagement). The implementation of the MDPs did not replace regular classroom instruction nor was it a replacement for existing practice. Rather, it served as an additional layer that teachers could interweave into their pedagogical approaches as they engaged in teaching their regular science curriculum. As in Phase 2, extensive data from teachers and students were collected.

Of note, as an early-stage design and development project based in a CDS perspective, M-PLANS did not include formal “control” conditions. Rather, the focus was on examining how the PL translated into teachers’ use of motivationally supportive instructional practices and how these practices were in turn associated with positive changes in student outcomes including their post-unit motivation, engagement during the implementation unit, and science learning during the unit. Below, we discuss the analysis of changes in teacher beliefs and enactment of M-PLANS aligned instructional practices and highlight key findings from analyses of student outcomes.

### M-PLANS: Teachers’ Beliefs and Instructional Practices

Results from lesson observations, interviews, focus groups, and pre-/post-implementation teacher surveys suggest that teachers perceived that the M-PLANS PL influenced their knowledge about and use of instructional practices for supporting student motivation and their related beliefs about motivation (Harris et al., 2023). Teachers reported that implementing the MDPs was both feasible and impactful. For instance, one teacher noted, “By thinking about the principles of motivation, I can start to make minor adjustments to my lesson plans that make a big difference in motivating students.” Importantly, teachers reported that the M-PLANS planning materials helped them motivate and engage all their students, “including [their] hard-to-reach students.”

A more in-depth analysis of data from the teachers who participated in Phase 3 showed evidence of alignment between key activities from the PL and the practices that teachers discussed in their post-implementation interviews (Mouzaoir et al., 2023). For instance, one teacher discussed how they supported the relevance MDP by connecting what they were learning in class about chemicals to the use of soap to clean their hands. Another sought to support autonomy by giving students choices in how to organize information about different chemicals by figuring out which dimensions (e.g., color, number of atoms, etc.) should form the basis of their proposed organization. This analysis also suggested, however, that teachers struggled to enact some of the MDP-supporting instructional strategies, such as how to provide meaningful (rather than superficial) choice, and that not all teachers perceived their MDP-aligned instructional practices as successful. Moreover, entering teacher beliefs may have limited teachers’ willingness to enact the MDPs as intended. For instance, some teachers continued to hold beliefs that not all students could “handle” heightened autonomy and that too much autonomy would “lead to chaos and misconceptions.”

Moving beyond teacher beliefs, the M-PLANS team also used classroom video data to investigate how teachers enacted the MDPs. Qualitative analyses of these videos indicated that teachers used a variety of strategies aligned with the M-PLANS PL, providing additional support for the effectiveness of the PL in shaping specific instructional practicfes (Cabrera et al., 2024; Liu et al., 2023, 2026; Shin, 2025). Notably, the specific ways the MDPs were enacted varied substantially across lessons and instructors. For instance, in Liu et al.’s (2023) case study of three co-design teachers from Phase 2, there was variability in how teachers supported students’ autonomy with respect to classroom management versus more cognitive aspects of learning. The variations seemed to reflect differences in teachers’ comfort with the subject area, with teachers with deeper chemistry knowledge providing additional supports for cognitive autonomy, as well as variability in teacher’s readiness and willingness to shift their classroom structures and management practices. Moreover, there was variability within classrooms in students’ perceptions of teachers’ motivational supports. In this way, the flexibility of the PL approach, which enabled teachers to drive the decision of when and how they enacted the MDPs, allowed teachers to draw on their strengths, consider the context, and account for their students’ strengths to develop a comprehensive, flexible, and context-driven approach to teacher instructional decision-making. However, it also highlighted the need for on-going support for teachers to help them broaden the ways they supported the MDPs in their classroom, potentially pushing them outside their comfort zones. Thus, one key takeaway from both the interview and observation data was that teachers likely need additional support and longer periods of enactment to help them overcome some of the challenges of implementation.

### M-PLANS PL: Student Outcomes

A key goal of M-PLANS was to support positive changes in students’ motivation. As such, the team was interested in how the enactment of the five MDPs together supported multiple motivational beliefs. Importantly, the M-PLANS team had hypothesized that it was the synergistic implementation of the MDPs that would be beneficial for student motivation, as multiple MDPs were thought to work together to support key motivational beliefs such as task values, competence beliefs, and goal orientations. Thus, to investigate how the MDPs combined to support students’ motivation, Kim et al. (2024) used latent profile analysis (LPA) to create clusters of perceived teacher use of instructional strategies aligned with the MDPs using data from the Phase 3 student post-unit survey. Their results suggested that a majority of students were most likely to belong to profiles associated with moderate to high perceived teacher instructional support aligned with the MDPs. Moreover, some of the variability seemed to reflect students’ individual differences, with a smaller group of students distributed across classes falling into some of the less common (and less supportive) profiles. MDP profile membership was in turn associated with students’ post-unit motivation, after controlling for pre-unit student motivation. These results suggest that when students perceived their teachers as using instructional practices aligned with the MDPs, there were benefits in terms of students’ own motivation.

The M-PLANS team also used students’ end-of-class reports to examine whether students’ perceptions of their teachers’ enactment of the MDPs were associated with students’ engagement. Correlational analyses of data from Phase 3 suggested that students’ perceptions of their teachers’ MDP-aligned instructional strategies were significantly correlated with students’ self-reported engagement. Students’ science learning was also assessed using a researcher-developed Knowledge-in-Use (KiU) assessment measuring students’ understanding of key scientific concepts covered in the chemistry units. After controlling for students’ initial motivation, results indicated that students who perceived that their teachers used instructional strategies aligned with the Confidence MDP had significantly higher science knowledge, as measured by the KiUs. There were no other significant differences for the other MDPs.

### Summary

In summary, the analyses conducted by the M-PLAN team suggest that teachers who participated in the M-PLANS PL shifted in both their beliefs about student motivation and the instructional strategies they used to support students’ motivation (Cabrera et al., 2024; Harris et al., 2023; Kim et al., 2024; Liu et al., 2023, 2026; Marchand et al., 2022; Mouzaoir et al., 2023; Shin, 2025). The results further revealed the interconnectedness among the MDPs themselves and the domain (in this case science) in which they were enacted, highlighting the importance of situating motivational supports within specific domains and contexts. Finally, there was evidence that students’ perceptions of MDP-aligned instruction varied both between and within classrooms, and that the perceived enactment of the MDPs was associated with students’ in-the-moment engagement and post-unit motivation. Of note, some of the within classroom variability was explained by students’ own initial motivational beliefs (see for instance Liu et al., 2023), highlighting the need to consider the complex interplay among the teachers’ instructional practices and the context as reflected by both students’ initial motivation and the subject domain. It also important to note that the evidence gathered to evaluate M-PLANS thus far was through an early-stage design-and-development project; it will be critical to gather additional, more robust evidence of the effectiveness of the M-PLANS PL both in supporting shifts in teachers’ beliefs and instructional practices as well as downstream consequences for students’ motivation, engagement, and learning.

## The Challenges and Promises of Conducting Integrative and CDS-Based Motivation Interventions

CORI and M-PLANS represent two attempts to implement theoretically integrated, multifaceted motivation interventions through deep involvement with educational practitioners and embedded changes in instructional practices and classroom contexts. While CORI was not based in a CDS framework per se, many of the decisions the researchers made in designing and implementing it are consistent with CDS views. For M-PLANS, a CDS perspective framed both the conceptualization of the intervention as well as the analytic approach. Using both of these examples as a backdrop, we now look to the future by discussing what we see as both some of the challenges of doing interventions like CORI and M-PLANS and also their promise for enhancing students’ motivation.

### Challenge 1: Indeterminacies in Understanding How and Why Interventions Work

One challenge with integrative and CDS-based approaches to motivation interventions is that there are both empirical and theoretical indeterminacies. Perhaps the most important is that working on multiple motivational principles with multiple teachers brings many indeterminacies into how results of each program can be explained. For instance, in explaining CORI’s results, why exactly did students’ reading comprehension and motivation improve? Were one or two of the motivational instructional principles particularly effective? Was it a result of adding the motivation enhancing principles to the strategy instruction? Or did the motivational principles and quality strategy instruction combine in ways that either one on its own would not have done (Guthrie et al. 2004a, b, provide an interesting discussion of these possibilities in light of the first CORI study’s results).

### Challenge 2: Do Multifaceted, Integrated Interventions Work the Same for All Students?

A second concern is whether or not the type of integrative, CDS interventions we are discussing here allow researchers to consider whether the interventions work in the same way for all students. In linear models, one might examine this by considering whether students with certain levels of prior achievement, demographic characteristics, or initial motivation respond in the same or different ways to the intervention and then use this information to decide which intervention might work best for different groups of students. However, a CDS-perspective suggests that one cannot easily break a system down into its subcomponents. Instead, it is important to consider more broadly the interplay of individual characteristics with the intervention in the context in which it is implemented, suggesting that interactions in linear models may not be sufficient to tease out the unique affordances and constraints of an intervention.

### Challenge 3: Implementation Autonomy

A third issue/potential critique is that embedded interventions that seek to change pedagogical approaches or curricula often provide teachers with a great deal of autonomy in how they implement the programs. Within both CORI and M-PLANS, the research teams embraced teachers as pedagogical experts who were best situated to determine how and when to implement the instructional design principles within their own context. As a result, there was quite a bit of variability in how teachers enacted the CORI and M-PLANS instructional approaches. These approaches challenge traditional notions of fidelity of treatment and further highlight the importance of framing interventions with a CDS lens. Indeed, the M-PLANS team found evidence that there were multiple ways in which teachers successfully enacted the MDPs (Cabrera et al., 2024; Liu et al., 2023; Shin, 2025). The team considered this a success of the project – rather than a problem.

### Promise 1: Attempting to Isolate Causes Misses the Point of these Interventions

We acknowledge that our proposed approach does indeed make it challenging to isolate specific causes given the interplay of factors contributing to the success of the intervention. However, as noted earlier, Guthrie et al. (2004a, b), pushed back on this notion that one must isolate specific causes in discussing the results of the elementary school studies. They stated that the CORI students’ better reading comprehension likely was due to the complex interplay of students’ increased motivation and greater strategy use and that attempting to isolate which of the motivational practices may be most influential is not worth doing in classroom settings because the practices depend on one another and on the classroom context. Indeed, they at one point attempted to isolate the components of their intervention by having teachers implement certain parts and found that doing so was not fruitful, likely because classroom practices designed to foster different aspects of motivation worked together in ways that could not be isolated.

Our view that the goal of integrated interventions is not to isolate specific causal mechanisms is consistent with Hulleman and Barron’s (2016) discussion as well, who note in reference to their discussion of the Carnegie Community College Intervention, “Because this is a multicomponent intervention, it is difficult to isolate which factors are primarily responsible for the improvement. But that’s not the focus of this effort. Instead, the most important goal is addressing the persistence problem.” (p. 168). Taking this one step further, one must ask what the main purpose of intervention research is. Is it to try to isolate specific causes? Or is the purpose to improve outcomes for as many students as possible, even if the exact reasons for the improvement cannot be clearly determined? As we discuss below (see Promise #2), moving away from isolating mechanisms does not mean that researchers should not attempt to understand whether, why, and how the intervention works. However, it does push against traditional, experimental approaches for doing so, as we discuss in greater detail below.

### Promise 2: New Ways of Assessing Effectiveness of Interventions

As researchers move towards embedded, CDS-based interventions, they may find that design-based implementation approaches are better suited than randomized control trials (RCTs) for assessing their effectiveness, especially in the early-stage design-and-development phases (see Penuel & Frank, 2016 for a discussion of these two approaches to educational research). Part of the concern with RCTs is that they often seek to identify universal principles that hold across contexts. However, a key premise of CDS is that if one conceptualizes education as a complex system, then one must adapt to local conditions and acknowledge the role of local conditions in how the intervention plays out in that setting (Kaplan et al., 2020; Lemke & Sabelli, 2008). This creates a real problem if researchers are only interested in effects that can be observed universally across contexts. Not taking into account unique local conditions can lead to real harm to students, not to mention the waste of resources. This idea is reflected in Penuel et al.’s (2011) discussion of design-based implementation research:

In design-based implementation research, the question may sometimes be one of “what works,” in which case experimental design may be appropriate. Instead, much design-based implementation approaches ask questions such as “What works when, how, and for whom? “How do we improve this reform strategy to make it more sustainable?” And “What capacities does the system need to continue to improve?” Answering these questions and the many subquestions needed to develop and validate theory-based innovations will require a wide range of methods. Longitudinal, historical, ethnographic, and case analyses of changing context are likely to be necessary to understand how reforms’ trajectories across time and setting shape implementation. (p. 335).

As an example (and in contrast to CORI), M-PLANS did not use a control group but instead utilized a “design and development” approach focused on examining how the professional development process impacted teachers’ instructional practices, and then students’ motivation. As we discussed in greater detail above, the M-PLANS team evaluated the effectiveness of the intervention using a variety of methods, both quantitative and qualitative, in which the researchers sought to document shifts in both teacher practices and students’ beliefs as emergent, dynamic processes. This approach allowed the team to account for the aggregation of interactions within the system as well as localized variations.

One goal when evaluating CDS-based interventions is to identify recurring emergent patterns and the circumstances that support them to anticipate that certain types of interventions will yield certain outcomes (Kaplan & Garner, 2020). As such, a CDS approach allows for the uniqueness of students’ motivation and teachers’ implementation of instructional supports (Challenges #2 and 3). Indeed, the variability observed in teachers’ implementation of the MDPs in M-PLANS provided guidance to the team about the areas where teachers might need more support (given variability in quality) whereas other forms of variability reflected unique enactments in the context of the classroom. Similarly, the use of profile analysis to identify groupings of students who perceived their teachers’ enactment of the MDPs in various ways both within and across classrooms supported the team’s ability to identify distinct patterns in classrooms where teachers effectively implement the MDPs versus those where they came close but struggled either because the science content was not being taught as deeply or because they struggled to create a warm climate (see Kim et al., 2024). Thus, we encourage motivational researchers to learn more about CDS and to consider how its application might help to advance the field’s understanding of motivation functions in situ. To be clear, we are not suggesting that experimental designs (such as RCTs or the switching replications design used in CORI) should be abandoned but are rather suggesting that RCTs are one among many viable research designs for evaluating interventions. And, some of these other methodologies are designed to provide more nuanced insights into the mechanisms that produce the effects of an intervention and focus greater attention on what matters for effective implementation. Thus, we caution against viewing experimental designs as the only *gold standard* and instead encourage researchers to consider them as one of many approaches that can be used to provide high quality evidence regarding the effectiveness of an intervention. This is another way we are calling for a paradigm shift - not just in how we approach intervention design, but in what research designs are used to assess their effectiveness.

Relatedly, it is quite possible to investigate underlying processes that are part of complex systems. However, the methodology for doing so varies considerably from the types of linear models often employed in educational psychology. For instance, Turner et al. (2014) used state-space grids to analyze the instructional practices of middle school teachers who participated in professional development around supporting students’ motivation across three years. They found that some teachers showed an upward trajectory in their use of the instructional strategies such that the teachers increased in their attempts to engage students over time and students responded in increasingly positive ways. For other teachers, however, students did not respond positively to the teachers’ attempts to support students’ engagement and teachers declined in their use of these supports over time. Turner et al.’s (2014) use of state-space grids to identify patterns of teacher-student interactions over three years clearly illustrates how one can come to understand the dynamic interplay between teachers and students as teachers attempted to implement the motivational supports they learned in the professional development. State-space grids are just one example of the various techniques that researchers might use to investigate motivational interventions using a CDS approach. Others include ethnography, orbital decomposition, social network analysis, and multivariate time series and recurrence quantification analysis, to name a few (see Koopmans, 2020 for a more detailed discussion).

A key idea in the approaches noted above is to acknowledge and investigate variability in how the intervention functions within complex systems (Kaplan et al., 2020). This requires a shift from approaches that aggregate across individuals such as (M)ANOVA, multiple regression, and many other approaches that seek to find aggregate statistical linear relations (Kaplan, 2023). Indeed, the observed heterogeneity in effects becomes the focus rather than error to be handled in one’s analysis.

Of note, this type of evaluative approach need not only apply to the type of embedded, theoretically integrated multifaceted interventions that were developed in CORI and M-PLANS: researchers studying targeted interventions such as utility-value interventions could employ similar statistical techniques. However, we contend that for researchers interested in transforming educational systems, it is critical to engage with stakeholders within that system to understand their needs and to work together to identify interventions that best fit the needs of those stakeholders (see also Sabelli & Harris, 2015). In some cases, targeted interventions may best serve the needs of those stakeholders, whereas in others the type of embedded, multifaceted interventions we developed in CORI and M-PLANS may be better suited.

A final note, design-based implementation research takes time. Interventions such as CORI and M-PLANS involved substantial engagement with community stakeholders and iterations to refine and shift the intervention to meet the needs of those stakeholders. Moreover, attempts to shift teacher beliefs and pedagogy are challenging, and may require many attempts and supports as teachers work to shift their understanding and ability to implement that understanding in their practice. As such, funding cycles may need to shift to allow for both longer-term partnerships and evaluations as well as to assess the long-term impacts of these approaches (Penuel et al., 2011).

So, what do you the reader think? Are these approaches to developing and assessing multifaceted interventions convincing to you? We acknowledge that at this point they show promise rather than being fully developed or articulated. We also acknowledge that our stance that multifaceted, embedded interventions should be the wave of the future needs to be documented by results showing this is the case; do such interventions have stronger short- and long-term effects than targeted interventions, at a time, effort, and resource point that is feasible? We do not yet have complete evidence on these kinds of outcomes yet, nor a full sense of the costs.

### A Final Challenge- and A Promising Response to it

With respect to the cost point, we would like to say more about the critique that multifaceted motivation interventions are costly and difficult to implement (Rosenzweig et al., 2022). For instance, even if multiple forms of motivation can be supported by a single instructional approach, such as supporting autonomy, integrated interventions tend to be broader, asking teachers or other intervention implementers to shift multiple aspects of the educational context. Thus, the “ask” of integrated interventions has the potential to be larger, such that teachers are asked to make many changes in their teaching practices. Nevertheless, when the intervention necessitates that teachers fundamentally shift their understanding and beliefs, it may yield a more sustained orientation to teaching in contrast to interventions that are not integrated with system change (see Garner & Kaplan, 2019, 2021 for a similar discussion). In this way, we contend that the ask of embedded interventions such as CORI and M-PLANS is different, but potentially more transformative. Moreover, the ask might be better aligned with what schools desire. Rather than an add-on program that schools must find time to implement in addition to the mandated school curricula (similar to SEL interventions), embedded, theoretically integrated multifaceted motivation interventions may fit within professional development programs that schools are already implementing. For instance, the M-PLANS research team has been approached by several school districts who are interested in implementing M-PLANS *because* they see it as aligned with their existing approaches to professional development and their goals for student learning.

One final point about interventions that involve changing classroom practices and/or focus more on teachers. The examples we gave were from elementary and middle school interventions. We think it quite possible that these types of interventions can also be developed and implemented at the college level - and some indeed have. For instance, Jones et al. (2020) provided professional development for university faculty to change their instruction based on the MUSIC integrated motivation model. We are also aware of efforts at both of our institutions that involve efforts to shift faculty instructional practices based on motivational theory.

## Conclusion

The impetus for the special issue was to encourage scholars to break out of theoretical silos, taking an integrative approach. We have argued that the landscape of motivational research is so broad that a key first step is thinking about how and/or why to integrate motivational theories with respect to their implications for intervention. However, we also fully embrace the longer-term goal of extending this view of students’ motivation as part of a complex dynamic system that must be considered together, rather than in theoretical silos, with research on self-regulation, metacognition, and personality. We suggest that as researchers consider these key questions of integration, both within motivation theories as well as in extension to self-regulation, metacognition, and personality and that they consider framing their work in alignment with CDS. Doing so is likely to yield a more comprehensive understanding of how educational contexts shape these key processes and how and where to intervene - as well as to be more likely to support the needs of more students in the classroom.

## Footnotes

1 Some of the multifaceted interventions we discuss here could also be referred to as instructional programs rather than interventions. Indeed, CORI is sometimes referred to as an instructional program and M-PLANS as a professional learning program. However, we think it is useful to frame this work more broadly as one type of intervention, thus we use the term “intervention” to describe both targeted and multifaceted interventions together as approaches for supporting students’ motivation.

2 Below, the five MDPs are described in the order used by the M-PLANS team, which the team re-ordered to follow the mnemonic acronym “Be CLeAR” (Belonging, Confidence, Learning Orientation, Autonomy, Relevance) as a memory aid for participating teachers.

3 The terms ‘design research’ approach (Collins et al., 2004) or ‘design experiment’ approach (Cobb et al., 2003; Bell, 2004) are also interchangeably used to refer to this type of research design. The approach of the M-PLANS team was also consistent with Penuel et al.’s (2011) description of design-based implementation research, given its focus on motivation as a persistent problem of practice identified by stakeholders, the use of a collaborative, iterative design approach, the focus on developing theory related to motivation and engagement as well as teacher professional learning, and the goal to develop an intervention that acknowledged local systems elements but that was also flexible enough to sustain change across multiple systems.

## Declarations

All ethical standards have been followed in producing this manuscript. Projects CORI and M-PLANS were approved by the institutional review boards of the authors’ institutions.

## References

[CR1] Ames, C. (1992). Classrooms: Goals, structures, and student motivation. *Journal of Educational Psychology*, *84*(3), 261–271. 10.1037/0022-0663.84.3.261

[CR2] Anderman, E. M. (2020). Achievement motivation theory: Balancing precision and utility. *Contemporary Educational Psychology,**61*, Article 101864. 10.1016/j.cedpsych.2020.101864

[CR3] Asher, M. W., Harackiewicz, J. M., Beymer, P. N., Hecht, C. A., Lamont, L. B., Else-Quest, N. M., Priniski, S. J., Thoman, D. B., Hyde, J. S., & Smith, J. L. (2023). Utility-value intervention promotes persistence and diversity in STEM. *Proceedings of the National Academy of Sciences,**120*(19), Article e2300463120. 10.1073/pnas.2300463120 10.1073/pnas.2300463120 PMC10175781 37126675

[CR4] Assor, A., Kaplan, H., & Roth, G. (2002). Choice is good, but relevance is excellent: Autonomy-enhancing and suppressing teacher behaviours predicting students’ engagement in schoolwork. *British Journal of Educational Psychology,**72*(2), 261–278. 10.1348/000709902158883 12028612 10.1348/000709902158883

[CR5] Bandura, A. (1986). Social foundations of thought and action. *Englewood Cliffs, NJ*, *1986*.

[CR6] Bandura, A. (1997). *Self-efficacy: The exercise of control*. W. H. Freeman.

[CR7] Barger, M. M., & Linnenbrink-Garcia, L. (2017). Developmental systems of students’ personal theories about education. *Educational Psychologist,**52*, 63–83. 10.1080/00461520.2016.1252264

[CR8] Baumeister, R. F., & Leary, M. R. (1995). The need to belong: Desire for interpersonal attachments as a fundamental human motivation. *Psychological Bulletin,**117*(3), 497–529. 10.1037/0033-2909.117.3.497 7777651

[CR9] Beachboard, M. R., Beachboard, J. C., Li, W., & Adkison, S. R. (2011). Cohorts and relatedness: Self-determination theory as an explanation of how learning communities affect educational outcomes. *Research in Higher Education,**52*, 853–874. 10.1007/s11162-011-9221-8

[CR10] Benden, D. K., & Lauermann, F. (2022). Students’ motivational trajectories and academic success in math-intensive study programs: Why short-term motivational assessments matter. *Journal of Educational Psychology,**114*(5), 1062–1085. 10.1037/edu0000708

[CR11] Beymer, P. N., Kim, Y., Allen, E. C., & Rosenzweig, E. Q. (2025). Examining a weekly cost reduction intervention in calculus. *Learning and Instruction,**100*, Article 102211.

[CR12] Blackwell, L. S., Trzesniewski, K. H., & Dweck, C. S. (2007). Implicit theories of intelligence predict achievement across an adolescent transition: A longitudinal study and an intervention. *Child Development,**78*(1), 246–263. 10.1111/j.1467-8624.2007.00995.x 17328703 10.1111/j.1467-8624.2007.00995.x

[CR13] Blumenfeld, P. C., Kempler, T. M., & Krajcik, J. S. (2006). Motivation and cognitive engagement in learning environments. In R. K. Sawyer (Ed.), *The Cambridge handbook of the learning sciences* (pp. 475–488). Cambridge University Press.

[CR14] Bråten, I., & Olaussen, B. S. (2005). Profiling individual differences in student motivation: A longitudinal cluster-analytic study in different academic contexts. *Contemporary Educational Psychology*, *30*(3), 359–396. 10.1016/j.cedpsych.2005.01.003

[CR15] Cabrera, L., Schmidt, J. A., Mouzaoir, S., Harris-Thomas., B., Conklin, K., Van Luven, W., Kim, E., Marchand, G. C., Harris, C. J., Linnenbrink-Garcia, L., & April (2024). Describing teachers’ instructional supports for students’ motivation: A qualitative approach. Paper presented at the annual meeting of the American Educational Research Association, Philadelphia, PA, United States.

[CR16] Carlone, H. B., & Johnson, A. (2007). Understanding the science experiences of successful women of color: Science identity as an analytic lens. *Journal of Research in Science Teaching,**44*(8), 1187–1218. 10.1002/tea.20237

[CR17] Cobb, P., Confrey, J., diSessa, A., Lehrer, R., & Schauble, L. (2003). Design experiments in educational research. *Educational Researcher,**32*(1), 9–13. 10.3102/0013189X032001009

[CR18] Cohen, G. L., Garcia, J., Apfel, N., & Master, A. (2006). Reducing the racial achievement gap: A social-psychological intervention. *Science,**313*, 1307–1310. 10.1126/science.1128317 16946074 10.1126/science.1128317

[CR19] Collins, A., Joseph, D., & Bielaczyc, K. (2004). Design research: Theoretical and methodological issues. *The Journal of the Learning Sciences*, *13*(1), 15–42. 10.1207/s15327809jls1301_2

[CR20] Conley, A. M. (2012). Patterns of motivation beliefs: Combining achievement goal and expectancy-value perspectives. *Journal of Educational Psychology*, *104*(1), 32–47. 10.1037/a0026042

[CR21] Davis, B., & Sumara, D. (2006). *Complexity and education: Inquiries into learning, teaching, and research*. Routledge.

[CR22] Davis, M. H., & Tonks, S. (2004). Diverse texts and technology for reading. In J. T. Guthrie, A. Wigfield, & K. Perencevich (Eds.), *Motivating reading comprehension: Concept Oriented Reading Instruction* (pp. 143–171). Erlbaum.

[CR23] Design-Based Research Collective. (2003). Design-based research: An emerging paradigm for educational inquiry. *Educational Researcher,**32*(1), 5–8. 10.3102/0013189x032001005

[CR24] Dietrich, J., Viljaranta, J., Moeller, J., & Kracke, B. (2017). Situational expectancies and task values: Associations with students’ effort. *Learning and Instruction*, *47*, 53–64. 10.1016/j.learninstruc.2016.10.009

[CR25] Dweck, C. S. (1999). *Self-theories: Their role in motivation, personality, and development*. Psychology. 2130257

[CR26] Dweck, C. S. (2017). From needs to goals and representations: Foundations for a unified theory of motivation, personality, and development. *Psychological Review*, *124*(6), 689–719. 10.1037/rev0000082 28933872 10.1037/rev0000082

[CR27] Eccles, J. S., & Wigfield, A. (2020). From expectancy-value theory to situated expectancy-value theory: A developmental, social cognitive, and sociocultural perspective on motivation. *Contemporary Educational Psychology,**61*, Article 101859. 10.1016/j.cedpsych.2020.101859

[CR28] Eccles, J. S., & Wigfield, A. (2024). The development, testing, and refinement of Eccles, Wigfield, and colleagues’ Situated Expectancy-Value model of achievement performance and choice. *Educational Psychology Review,**36*(2), Article 51. 10.1007/s10648-024-09888-9

[CR29] Eccles, J. S., Adler, T. F., Futterman, R., Goff, S. B., Kaczala, C. M., Meece, J. L., Midgley, C., & Spence, J. T. (Eds.). (1983). Achievement and achievement motivation (75–146). W. H. Freeman.

[CR30] Eccles, J. S., Midgley, C., Wigfield, A., Buchanan, C. M., Reuman, D., & Flanagan, C. (1993). The impact of stage-environment on young adolescents’ experiences in schools and in families. *American Psychologist,**48*(2), 90–101. 10.1037/0003-066x.48.2.90 8442578 10.1037//0003-066x.48.2.90

[CR31] Fishbach, A., & Ferguson, M. J. (2007). The goal construct in social psychology. *In A. W. Kruglanski & E. T. Higgins (Eds.), Social psychology: Handbook of basic principles (2nd ed., pp. 490–515). The Guilford Press.*

[CR32] Furrer, C., & Skinner, E. (2003). Sense of relatedness as a factor in children’s academic engagement and performance. *Journal of Educational Psychology*, *95*(1), 148–162. 10.1037/0022-0663.95.1.148

[CR33] Garner, J. K., & Kaplan, A. (2019). A complex dynamic systems perspective on teacher learning and identity formation: An instrumental case. *Teachers and Teaching*, *25*(1), 7–33. 10.1080/13540602.2018.1533811

[CR34] Garner, J. K., & Kaplan, A. (2021). A complex dynamic systems approach to the design and evaluation of teacher professional development. *Professional Development in Education,**47*(2–3), 289–314. 10.1080/19415257.2021.1879231

[CR35] Gaspard, H., Häfner, I., Parrisius, C., Trautwein, U., & Nagengast, B. (2017). Assessing task values in five subjects during secondary school: Measurement structure and mean level differences across grade level, gender, and academic subject. *Contemporary Educational Psychology*, *48*, 67–84. 10.1016/j.cedpsych.2016.09.003

[CR36] Geerling, D., Butner, J., Fraughton, T., Sinclair, S., Zachary, J., & Sansone, C. (2020). The dynamic association of interest and confusion: The potential for moderation by utility value and gender. *The Journal of Experimental Education*, *88*(3), 407–430. 10.1080/00220973.2018.1561403

[CR37] Graham, S. (2020). An attributional theory of motivation. *Contemporary Educational Psychology,**61*, Article 101861. 10.1016/j.cedpsych.2020.101861

[CR38] Guskey, T. R. (2014). Measuring the effectiveness of educators’ professional development. In L. E. Martin, S. Kragler, D. J. Quatroche, & K. L. Bauserman (Eds.), *Handbook of professional development in education: Successful models and practices, preK-12* (pp. 447–466). Guilford Press.

[CR39] Guthrie, J. T., & Klauda, S. L. (2014). Effects of classroom practices on reading comprehension, engagement, and motivations for adolescents. *Reading Research Quarterly,**49*(4), 387–416. 25506087 10.1002/rrq.81 PMC4264840

[CR40] Guthrie, J. T., Wigfield, A., & VonSecker, C. (2000). Effects of integrated instruction on motivation and strategy use in reading. *Journal of Educational Psychology*, *92*(2), 331–341. 10.1037/0022-0663.92.2.331

[CR41] Guthrie, J. T., Wigfield, A., Barbosa, P., Perencevich, K. C., Taboada, A., Davis, M. H., Scafiddi, N. T., & Tonks, S. (2004a). Increasing reading comprehension and engagement through concept-oriented reading instruction. *Journal of Educational Psychology,**96*(3), 403–423. 10.1037/0022-0663.96.3.403

[CR42] Guthrie, J. T., Wigfield, A., & Perencevich, K. (Eds.). (2004b). *Motivating reading comprehension: Concept Oriented Reading Instruction*. Lawrence Erlbaum Associates.

[CR43] Guthrie, J. T., McRae, A., & Klauda, S. L. (2007). Contributions of concept oriented reading instruction to knowledge about motivation efforts in reading. *Educational Psychologist,**42*(4), 237–250. 10.1080/00461520701621087

[CR44] Guthrie, J. T., Wigfield, A., & Klauda, S. L. (Eds.). (2012). *Adolescents’ engagement in academic literacy.* University of Maryland.

[CR45] Guthrie, J. T., Klauda, S. L., & Ho, A. N. (2013). Modeling the relationships among reading instruction, motivation, engagement, and achievement for adolescents. *Reading Research Quarterly,**48*(1), 9–26. 26412903 10.1002/rrq.035 PMC4583135

[CR46] Harackiewicz, J. M., & Priniski, S. J. (2018). Improving student outcomes in higher education: The science of targeted intervention. *Annual Review Psychology*, *69*, 409–435. 10.1146/annurev-psych-122216-011725 10.1146/annurev-psych-122216-011725 PMC6211287 28934586

[CR47] Harackiewicz, J. M., Canning, E. A., Tibbetts, Y., Priniski, S. J., & Hyde, J. S. (2016). Closing achievement gaps with a utility-value intervention: Disentangling race and social class. *Journal of Personality and Social Psychology*, *111*(5), 745–765. 10.1037/pspp0000075 26524001 10.1037/pspp0000075 PMC4853302

[CR48] Harris, C. J., & Allen, C. D. (2022). Project-Based Learning Environments Research. In T. L. Good, & M. McCaslin (Eds.), *), Routledge encyclopedia of education: Educational psychology*. Taylor & Francis. 10.4324/9781138609877-REE138-1

[CR49] Harris, C. J., Schmidt, J. A., Linnenbrink-Garcia, L., Marchand, G. C., Cabrera, L., McKinney, D., & Liu, P. (2023). *Equipping science teachers to support student motivation in NGSS classrooms: Insights from the development of the M-PLANS program*. WestEd.

[CR50] Hecht, C. A., Priniski, S., & Harackiewicz, J. M. (2019). Understanding long-term effects of motivation interventions in a changing world. In E. N. Gonida, & M. S. Lemos (Eds.), *Motivation in education at a time of global change: Theory, research and implications for practice* (Vol. 20, pp. 81–99). Emerald. (Advances in Motivation. 10.1108/S0749-742320190000020005 PMC6726402 31485099

[CR51] Heckhausen, J., & Heckhausen, H. (2018). Motivation and action: Introduction and overview. In J. Heckhausen & H. Heckhausen (Eds.), *Motivation and Action* (pp. 1–14). Springer International Publishing. 10.1007/978-3-319-65094-4_1

[CR52] Heino, M. T. J., Proverbio, D., Marchand, G., Resnicow, K., & Hankonen, N. (2023). Attractor landscapes: A unifying conceptual model for understanding behaviour change across scales of observation. *Health Psychology Review*, *17*(4), 655–672. 10.1080/17437199.2022.2146598 36420691 10.1080/17437199.2022.2146598 PMC10261543

[CR53] Hidi, S., & Renninger, K. A. (2006). The four phase model of interest development. *Educational Psychologist*, *41*(2), 111–127. 10.1207/s15326985ep4102_4

[CR54] Hilpert, J. C., & Marchand, G. C. (2018). Complex systems research in educational psychology: Aligning theory and method. *Educational Psychologist*, *53*(3), 185–202. 10.1080/00461520.2018.1469411 31431794 10.1080/00461520.2018.1469411 PMC6701846

[CR55] Hulleman, C. S., & Barron, K. E. (2016). Motivation interventions in education: Bridging theory, research, and practice. In L. Corno, & E. M. Anderman (Eds.), *Handbook of Educational Psychology, Third Edition* (pp. 160–171). Taylor and Francis.

[CR56] Hulleman, C. S., & Harackiewicz, J. M. (2009). Promoting interest and performance in high school science classes. *Science*, *326*(5958), 1410–1412. 10.1126/science.1177067 19965759 10.1126/science.1177067

[CR57] Jacobs, J., Lanza, S., Osgood, D. W., Eccles, J. S., & Wigfield, A. (2002). Ontogeny of children’s self-beliefs: Gender and domain differences across grades one through 12. *Child Development,**73*(2), 509–527. 10.1111/1467-8624.00421 11949906 10.1111/1467-8624.00421

[CR58] Jang, H., Reeve, J., & Halusic, M. (2016). A new autonomy-supportive way of teaching that increases conceptual learning: Teaching in students’ preferred ways. *The Journal of Experimental Education*, *84*(4), 686–701. 10.1080/00220973.2015.1083522

[CR59] Jones, B. D. (2009). Motivating students to engage in learning: The MUSIC Model of Academic Motivation. *International Journal of Teaching and Learning in Higher Education*, *21*(2), 272–285. https://www.isetl.org/ijtlhe/pdf/IJTLHE774.pdf

[CR60] Jones, B. D., Biscotte, S., & Becker, T. H. (2020). Using a motivation model and student data to redesign general education courses: An examination of a faculty development approach. *Journal of General Education*, *69*(3–4), 235–250. 10.5325/jgeneeduc.69.3-4.0235

[CR61] Jones, B. D., Ambarkutuk, Z., & Schunk, D. H. (2025). Applications of the MUSIC model of motivation and its associated inventories: A systematic review and meta-analysis. *International Journal of Applied Positive Psychology*, *10*, 67. 10.1007/s41042-025-00249-7

[CR62] Kaplan, A. (Ed.). (2023). Understanding human motivation and action as a complex dynamic system. In M. Bong, S-i. Kim, & J. Reeve (Eds.), Motivation Science: Controversies and Insights (pp. 448–452). Oxford University Press.

[CR63] Kaplan, A., & Garner, J. K. (2017). A complex dynamic systems perspective on identity and its development: The dynamic systems model of role identity. *Developmental Psychology,**53*(11), 2036–2051. 10.1037/dev0000339 29094968 10.1037/dev0000339

[CR64] Kaplan, A., & Garner, J. K. (2020). Steps for applying the complex dynamical systems approach in educational research: A guide for the perplexed scholar. *The Journal of Experimental Education,**88*(3), 486–502. 10.1080/00220973.2020.1745738

[CR65] Kaplan, A., Cromley, J., Perez, T., Dai, T., Mara, K., & Balsai, M. (2020). The role of context in educational RCT findings: A call to redefine “evidence-based practice.” *Educational Researcher,**49*(4), 285–288. 10.3102/0013189X20921862

[CR66] Kelly, G. (1955). *The psychology of personal constructs*. Norton.

[CR67] Kim, E., Linnenbrink-Garcia, L., Cabrera, L., McKinney, D., Schmidt, J. A., Harris, C. J., Marchand, G., & April (2024). *Student perceptions of teacher motivational support: Latent profile analysis.* Paper presented at the annual meeting of the American Educational Research Association, Philadelphia, PA, United States.

[CR68] Koopmans, M. (2020). Education is a complex dynamical system: Challenges for research. *The Journal of Experimental Education*, *88*(3), 358–374. 10.1080/00220973.2019.1566199

[CR69] Kosovich, J. J., Flake, J. K., & Hulleman, C. S. (2017). Short-term motivation trajectories: A parallel process model of expectancy-value. *Contemporary Educational Psychology,**49*, 130–139. 10.1016/j.cedpsych.2017.01.004

[CR70] Krajcik, J. S., Blumenfeld, P. C., Marx, R. W., & Soloway, E. S. (1994). A collaborative model for helping middle grade science teachers learn project-based instruction. *The Elementary School Journal,**94*(5), 483–497. 10.1086/461779

[CR71] Ladson-Billings, G. (1995). Toward a theory of culturally relevant pedagogy. *American Educational Research Journal*, *32*(3), 465–491. 10.3102/00028312032003465

[CR72] Lazowski, R. A., & Hulleman, C. S. (2016). Motivation interventions in education: A meta-analytic review. *Review of Educational Research*, *86*(2), 602–640. 10.3102/0034654315617832

[CR73] Lemke, J. L., & Sabelli, N. H. (2008). Complex systems and educational change: Towards a new research agenda. *Educational Philosophy and Theory,**40*(1), 118–120. 10.1111/j.1469-5812.2007.00401.x

[CR74] Linnenbrink-Garcia, L., Patall, E. A., & Pekrun, R. (2016). Adaptive motivation and emotion in education: Research and principles for instructional design. *Policy Insights from the Behavioral and Brain Sciences*, *3*(2), 228–236. 10.1177/2372732216644450

[CR75] Liu, P., McKinney, D., Lee, A. A., Schmidt, J. S., Marchand, G. C., & Linnenbrink-Garcia, L. (2023). A mixed-methods exploration of mastery goal support in 7th-grade science classrooms. *Cognition and Instruction*, *41*(2), 201–247. 10.1080/07370008.2022.2140807

[CR76] Liu, P., Marchand, G. C., Harris, C. J., Schmidt, J. A., & Linnenbrink-Garcia, L. (2026). Patterns of agentic engagement, autonomy support, and extrinsic motivation in middle school science. *Journal of Educational Psychology. *(In press).

[CR77] Mac Iver, D. J., Stipek, D. J., & Daniels, D. H. (1991). Explaining within-semester changes in student effort in junior high school and senior high school courses. *Journal of Educational Psychology,**83*(2), 201–211. 10.1037/0022-0663.83.2.201

[CR78] Maehr, M. L., & Midgley, C. (1996). *Transforming school cultures*. Westview.

[CR79] Maehr, M., L., & Zusho, A. (2009). Achievement goal theory: The past, present, and future. *Handbook of Motivation at School*. Routledge.

[CR80] Magnusson, D., & Cairns, R. B. (1996). Developmental science: Toward a unified framework. In R. B. Cairns, G. H. Elder, & E. J. Costello (Eds.), *Developmental Science* (pp. 7–30). Cambridge University Press.

[CR81] Marchand, G. C., & Hilpert, J. C. (2020). Complex systems approaches to educational research: Introduction to the special issue. *The Journal of Experimental Education,**88*(3), 351–357. 10.1080/00220973.2020.1746625

[CR82] Marchand, G. C., & Hilpert, J. C. (2023). Contributions of complex systems approaches, perspectives, models, and methods in educational psychology. *Handbook of Educational Psychology* (4th ed.). Routledge.

[CR83] Marchand, G., Schmidt, J. A., Linnenbrink-Garcia, L., Harris, C. J., McKinney, D., & Liu, P. (2022). Lessons from a co-design team on supporting student motivation in middle school science classrooms. *Theory into Practice*, *61*(1), 113–128. 10.1080/00405841.2021.1932155

[CR84] Martin, A. J. (2007). Examining a multidimensional model of student motivation and engagement using a construct validation approach. *British Journal of Educational Psychology,**77*(2), 413–440. 10.1348/000709906X118036 17504555 10.1348/000709906X118036

[CR85] Martin, A. J. (2008). Enhancing student motivation and engagement: The effects of a multidimensional intervention. *Contemporary Educational Psychology*, *33*(2), 239–269. 10.1016/j.cedpsych.2006.11.003

[CR86] Martin, A. J., Papworth, B., Ginns, P., Malmberg, L. E., Collie, R. J., & Calvo, R. A. (2015). Real-time motivation and engagement during a month at school: Every moment of every day for every student matters. *Learning and Individual Differences*, *38*, 26–35. 10.1016/j.lindif.2015.01.014

[CR87] Middleton, M. J., Kaplan, A., & Midgley, C. (2004). The change in middle school students’ achievement goals in mathematics over time. *Social Psychology of Education*, *7*(3), 289–311. 10.1023/B:SPOE.0000037484.86850.fa

[CR88] Mouzaoir, S., Liu, P., Harris-Thomas, B., Lee, A., Conklin, K. M., McKinney, D., Harris, C. J., Linnenbrink-Garcia, L., Marchand, G. C., Schmidt, J. A., & April (2023). *Examining science teachers’ changes in practice following motivation-focused professional learning.* Paper presented at the annual meeting of the American Educational Research Association, Chicago, IL, United States.

[CR89] Muis, K. R., Ranellucci, J., Franco, G. M., & Crippen, K. J. (2013). The interactive effects of personal achievement goals and performance feedback in an undergraduate science class. *Journal of Experimental Education*, *81*(4), 556–578. 10.1080/00220973.2012.738257

[CR90] Murphy, P. K., & Alexander, P. A. (2000). A motivated exploration of motivation terminology. *Contemporary Educational Psychology*, *25*(1), 3–53. 10.1006/ceps.1999.1019 10620380 10.1006/ceps.1999.1019

[CR91] Musu-Gillette, L., Wigfield, A., Harring, J., & Eccles, J. S (2015). Trajectories of change in students’ self-concepts of ability and values in math and college major choice. *Educational Research and Evaluation*, *21*(4), 343–370. 10.1080/13803611.2015.1057161

[CR92] National Research Council. (2012). *A framework for k-12 science education: Practices, crosscutting concepts, and core ideas*. National Academies. 10.17226/13165

[CR93] NGSS Lead States. (2013). *Next generation science standards: For states, by states*. National Academies. 10.17226/18290

[CR94] Palmer, D. H. (2009). Student interest generated during an inquiry skills lesson. *Journal of Research in Science Teaching*, *46*(2), 147–165. 10.1002/tea.20263

[CR95] Parrisius, C., Gaspard, H., Zitzmann, S., Trautwein, U., & Nagengast, B. (2022). The situative nature of competence and value beliefs and the predictive power of autonomy support: A multilevel investigation of repeated observations. *Journal of Educational Psychology*, *114*(4), 791–814. 10.1037/edu0000680

[CR96] Patall, E. S. (2020). The complex role of choice in human motivation and functioning. In R. M. Ryan (Ed.), *Oxford handbook of motivation* (2nd. ed., pp. 135–156). Oxford University Press.

[CR97] Patall, E. A., Linnenbrink-Garcia, L., Liu, P., Zambrano, J., & Yates, N. (2026). Instructional practices that support adaptive motivation, engagement, and learning. In A. O’Donnell, N.C. Barnes, & J. Reeve (Eds.), *Oxford Handbook of Educational Psychology* (pp. 829–878)*.* Oxford Academic. 10.1093/oxfordhb/9780199841332.013.36

[CR98] Patrick, H., Kaplan, A., & Ryan, A., M (2011). Positive classroom motivational environments: Convergence between mastery goal structure and the classroom social climate. *Journal of Educational Psychology*, *103*(2), 367–382. 10.1037/a0023311

[CR99] Penuel, W. R., & Frank, K. (2016). Modes of inquiry in educational psychology and learning science research. In L. Corno, & E. M. Anderman (Eds.), *Handbook of Educational Psychology, Third Edition* (pp. 16–28). Taylor & Francis.

[CR100] Penuel, W. R., Fishman, B. J., Cheng, H., B., & Sabelli, N. (2011). Organizing research and development at the intersection of learning, implementation, and design. *Educational Researcher*, *40*(7), 331–337. 10.3102/0013189X11421826

[CR101] Perry, R. P., Stupnisky, R. H., Hall, N. C., Chipperfield, J. G., & Weiner, B. (2010). Bad starts and better finishes: Attributional retraining and initial performance in competitive achievement settings. *Journal of Social and Clinical Psychology*, *29*(6), 668–700. 10.1521/jscp.2010.29.6.668

[CR102] Pervin, L. (2015). *Goal concepts in personality and social psychology*. Taylor & Francis.

[CR103] Pintrich, P. R. (2003). A motivational science perspective on the role of student motivation in learning and teaching contexts. *Journal of Educational Psychology*, *95*(4), 667–686. 10.1037/0022-0663.95.4.667

[CR104] Pugh, K. J., Bergstrom, C. M., Heddy, B. C., & Krob, K. E. (2017). Supporting deep engagement: The Teaching for Transformative Experiences in Science (TTES) Model. *The Journal of Experimental Education*, *85*(4), 629–657. 10.1080/00220973.2016.1277333

[CR105] Renninger, K. A., & Hidi, S. (2016). *The power of interest for motivation and engagement*. Routledge.

[CR106] Robinson, K. A., Perez, T., Nuttall, A. K., Roseth, C. J., & Linnenbrink-Garcia, L. (2018). From science student to scientist: Predictors and outcomes of heterogeneous science identity trajectories in college. *Developmental Psychology*, *54*, 1977–1922. 10.1037/dev0000567 30234346 10.1037/dev0000567 PMC6152842

[CR107] Rohlwing, R. L., & Spelman, M. (2014). Characteristics of adult learning: Implications for the design and implementation of professional development programs. In L. E. Martin, S. Kragler, D. J. Quatroche, & K. L. Bauserman (Eds.), *Handbook of professional development in education: Successful models and practices, preK-12* (pp. 231–245). Guilford Press.

[CR108] Rosenzweig, E. Q., Wigfield, A., & Hulleman, C. S. (2020). More useful, or not so bad? Examining the effects of utility value and cost reduction interventions in college physics. *Journal of Educational Psychology*, *112*, 166–182. 10.1037/edu0000370

[CR109] Rosenzweig, E. Q., Wigfield, A., & Eccles, J. S. (2022). Beyond utility value interventions: The why, when, and how for next steps in expectancy-value intervention research. *Educational Psychologist*, *57*(1), 11–30. 10.1080/00461520.2021.1984242

[CR110] Ryan, R. M., & Deci, E. L. (2017). *Self-determination theory: Basic psychological needs in motivation, development, and wellness*. Guilford.

[CR111] Ryan, R. M., & Deci, E. L. (2020). Intrinsic and extrinsic motivation from a self-determination theory perspective: Definitions, theory, practices, and future directions. *Contemporary Educational Psychology*, *61*, 101860. 10.1016/j.cedpsych.2020.101860

[CR112] Sabelli, N. H., & Harris, C. J. (2015). The role of innovation in scaling up educational innovations. In C.-K. Looi & L. W. Teh (Eds.), *Scaling Educational Innovations* (pp. 13–30). Springer.

[CR113] Saqr, M., & López-Pernas, S. (2024). Mapping the self in self-regulation using complex dynamic systems approach. *British Journal of Educational Technology*, *55*(4), 1376–1397. 10.1111/bjet.13452

[CR114] Schunk, D. H., & DiBenedetto, M. K. (2020). Motivation and social cognitive theory. *Contemporary Educational Psychology*, *60*, 101832. 10.1016/j.cedpsych.2019.101832

[CR115] Shell, D. F., & Husman, J. (2008). Control, motivation, affect, and strategic self-regulation in the college classroom: A multidimensional phenomenon. *Journal of Educational Psychology*, *100*(2), 443–459. 10.1037/0022-0663.100.2.443

[CR116] Shin, S. H. (2025). *Understanding change in students’ competence beliefs: A mixed methods study* (Doctoral dissertation, Michigan State University). ProQuest Dissertations & Theses Global.

[CR117] Skinner, E. A., Rickert, N. P., Vollet, J. W., & Kindermann, T. A. (2022). The complex social ecology of academic development: A bioecological framework and illustration examining the collective effects of parents, teachers, and peers on student engagement. *Educational Psychologist*, *57*(2), 87–113. 10.1080/00461520.2022.2038603

[CR118] Slavin, R. E., & Madden, N. A. (2010). Success for All: prevention and early intervention in school-wide reform. *Handbook of Research on Schools, Schooling and Human Development*. Routledge.

[CR119] Slavin, R. E., Hurley, E. A., & Chamberlain, A. (2003). Cooperative learning and achievement: Theory and research. *Handbook of Psychology: Educational Psychology*, (*Vol. 7*, pp. 177–198). John Wiley & Sons, Inc. 10.1002/0471264385.wei0709

[CR120] Smith, L. B., & Thelen, E. (2003). Development as a dynamic system. *Trends in Cognitive Sciences*, *7*(8), 343–348. 10.1016/S1364-6613(03)00156-6 12907229 10.1016/s1364-6613(03)00156-6

[CR121] Soenens, B., & Vansteenkiste, M. (2005). Antecedents and outcomes of self-determination in three life domains: The role of parents’ and teachers’ autonomy support. *Journal of youth and adolescence*, *34*, 589–604. 10.1007/s10964-005-8948-y

[CR122] Stokes, D. (1997). *Pasteur’s quadrant: Basic science and technological innovation*. Brookings Institution.

[CR123] Tamura, A., Ishii, R., Yagi, A., Fukuzumi, N., Hatano, A., Sakaki, M., Tanaka, A., & Murayama, K. (2022). Exploring the within-person contemporaneous network of motivational engagement. *Learning and Instruction*, *81*, 101649. 10.1016/j.learninstruc.2022.101649

[CR124] Thelan, E. (2005). Dynamic systems theory and the complexity of change. *Psychoanalytic Dialogues*, *15*(2), 255–283. 10.1080/10481881509348831

[CR125] Trochim, W. M. K. (2005). *Research methods: The concise knowledge base*. Atomic Dog Publishing, Inc.

[CR126] Turner, J. C., Christensen, A., Kackar-Cam, H. Z., Trucano, M., & Fulmer, S. M. (2014). Enhancing students’ engagement: Report of a 3-year intervention with middle school teachers. *American Educational Research Journal*, *51*(6), 1195–1226. http://www.jstor.org/stable/24546715

[CR127] Urdan, T., & Kaplan, A. (2020). The origins, evolution, and future directions of achievement goal theory. *Contemporary Educational Psychology*, *61*, 101862. 10.1016/j.cedpsych.2020.101862

[CR128] Urhahne, D., & Wijnia, L. (2023). Theories of motivation in education: An integrative framework. *Educational Psychology Review*, *35*, 45. 10.1007/s10648-023-09767-9

[CR129] Usher, E. L. (2016). Personal capability beliefs. In L. Corno, & E. M. Anderman (Eds.), *Handbook of Educational Psychology* (3rd ed., pp. 146–159). Routledge/Taylor & Francis Group.

[CR130] Walton, G. M. (2014). The new science of wise psychological interventions. *Current Directions in Psychological Science*, *23*(1), 73–82. 10.1177/0963721413512856

[CR131] Walton, G. M., & Yeager, D. S. (2020). Seed and soil: Psychological affordances in contexts help to explain where wise interventions succeed or fail. *Current Directions in Psychological Science*, *29*(3), 219–226. 10.1177/0963721420904453 32719574 10.1177/0963721420904453 PMC7384695

[CR132] Weiner, B. (1985). An attributional theory of achievement motivation and emotion. *Psychological Review*, *92*(4), 548–573. 10.1037/0033-295X.92.4.548 3903815

[CR133] Weiner, B. (1990). History of motivational research in education. *Journal of Educational Psychology*, *82*(4), 616–622. 10.1037/0022-0663.82.4.616

[CR134] Weiner, B. (2010). The development of an attribution-based theory of motivation: A history of ideas. *Educational Psychologist,**45*(1), 28–36. 10.1080/00461520903433596

[CR135] Wigfield, A. (1994). Expectancy - Value theory of achievement motivation: A developmental perspective. *Educational Psychology Review,**6*(1), 49–78. 10.1007/BF02209024 10.1006/ceps.1999.1015 10620382

[CR136] Wigfield, A., & Koenka, A. C. (2020). Where do we go from here in academic motivation theory and research? Some reflections and recommendations for future work. *Contemporary Educational Psychology,**61*, Article 101872. 10.1016/j.cedpsych.2020.101872

[CR137] Wilkins, J. L. M., Jones, B. D., & Fenerci, H. (2025). Investigating the motivational climate of a mathematics education course as a complex dynamic system. *International Journal of Complexity in Education*, *6*(1). 10.26262/ijce.v6i1.10850

[CR138] Wilson, T. D., & Linville, P. W. (1982). Improving the academic performance of college freshmen: Attribution therapy revisited. *Journal of Personality and Social Psychology,**42*(2), 367–376. 10.1037/0022-3514.42.2.367

[CR139] Wolff, S. M., Hilpert, J. C., Vongkulluksn, V. W., Bernacki, M. L., & Greene, J. A. (2024). Self-efficacy inertia: The role of competency beliefs and academic burden in achievement. *Contemporary Educational Psychology,**79*, Article 102315. 10.1016/j.cedpsych.2024.102315

[CR140] Yeager, D. S., & Walton, G. M. (2011). Social-psychological interventions in education: They’re not magic. *Review of Educational Research,**81*(2), 267–301. 10.3102/0034654311405999

[CR141] Yeager, D. S., Hanselman, P., Walton, G. M., et al. (2019). A national experiment reveals where a growth mindset improves achievement. *Nature,**573*, 364–369. 10.1038/s41586-019-1466-y 31391586 10.1038/s41586-019-1466-y PMC6786290

